# Synthesis of New Brassinosteroid 24-Norcholane Type Analogs Conjugated in C-3 with Benzoate Groups

**DOI:** 10.3390/molecules26041173

**Published:** 2021-02-22

**Authors:** Karoll Ferrer, Katy Díaz, Miroslav Kvasnica, Andrés F. Olea, Mauricio Cuellar, Luis Espinoza

**Affiliations:** 1 Departamento de Química, Universidad Técnica Federico Santa María, Avenida España 1680, Valparaíso 2340000, Chile; karoll.ferrer.14@sansano.usm.cl (K.F.); katy.diaz@usm.cl (K.D.); 2 Laboratory of Growth Regulators, Institute of Experimental Botany, The Czech Academy of Sciences, Palacký University, Šlechtitelů 27, 78371 Olomouc, Czech Republic; 3 Department of Experimental Biology, Faculty of Science, Palacký University, Šlechtitelů 27, 78371 Olomouc, Czech Republic; 4 Instituto de Ciencias Químicas Aplicadas, Facultad de Ingeniería, Universidad Autónoma de Chile, El Llano Subercaseaux 2801, Santiago 8900000, Chile; andres.olea@uautonoma.cl; 5 Facultad de Farmacia, Escuela de Química y Farmacia, Universidad de Valparaíso, Av. Gran Bretaña 1093, Valparaíso 2340000, Chile; mauricio.cuellar@uv.cl

**Keywords:** synthesis, brassinosteroids, analogs, 24-norcholane, benzoate esters, Rice Lamina Inclination Test, conjugated in C-3

## Abstract

The metabolism of brassinosteroid leads to structural modifications in the ring skeleton or the side alkyl chain. The esterification and glycosylation at C-3 are the most common metabolic pathways, and it has been suggested that conjugate brassinosteroids are less active or inactive. In this way, plants regulate the content of active brassinosteroids. In this work, the synthesis of brassinosteroid 24-norcholane type analogs conjugated at C-3 with benzoate groups, carrying electron donor and electron attractant substituents on the aromatic ring, is described. Additionally, their growth-promoting activities were evaluated using the Rice Lamina Inclination Test (RLIT) and compared with that exhibited by brassinolide (used as positive control) and non-conjugated analogs. The results indicate that at the lowest tested concentrations (10^−8^–10^−7^ M), all analogs conjugated at C-3 exhibit similar or higher activities than brassinolide, and the diasteroisomers with *S* configuration at C-22 are the more active ones. Increasing concentration (10^−6^ M) reduces the biological activities of analogs as compared to brassinolide.

## 1. Introduction

Brassinosteroids (BRs) are an important group of polyhydroxylated sterol plant growth regulators in multiple developmental processes, at nanomolar to micromolar concentration, including cell division, cell elongation, vascular differentiation, reproductive development, and modulation of gene expression [1]. BRs also influence various other developmental processes such as the germination of seeds, rhizogenesis, flowering, senescence, abscission, and maturation. They also confer resistance to plants against various abiotic and biotic stresses [2,3,4,5].

**Figure 1:**
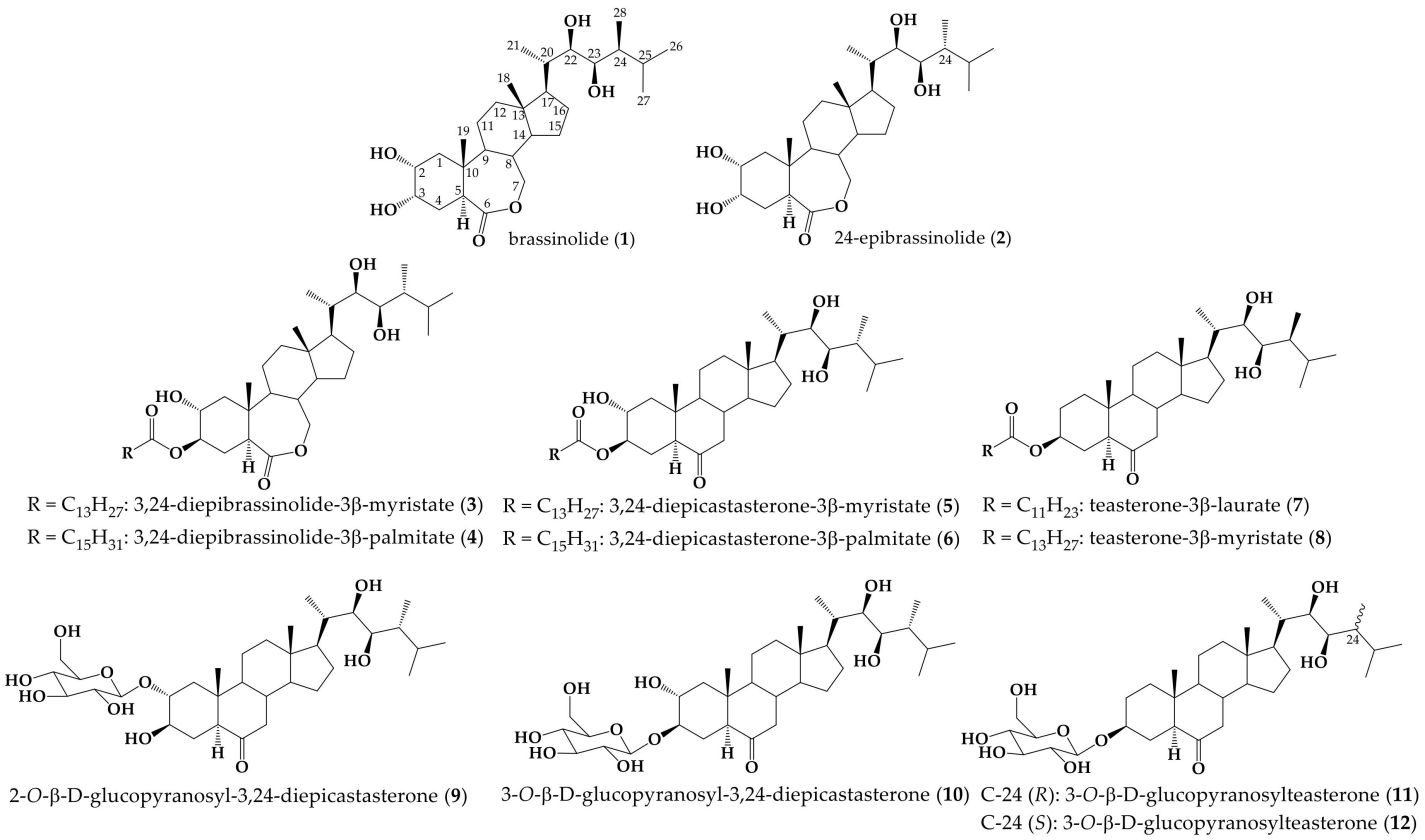
Structure of natural occurring brassinolide (**1**), 24-epibrasinolide (**2**), metabolites conjugated in C-3 formed by esterification (compounds **3**–**8**), and metabolites conjugated in C-2 and C-3 formed by glycosylation (compounds **9**–**12**).

Since the discovery of brassinolide (**1**) (Figure 1) [6], 70 BRs, among them 65 unconjugated (free) and 5 conjugated BRs, have been isolated from 60 plant species including 51 angiosperms (12 monocotyledons and 39 dicotyledons), 6 gymnosperms, 1 pteridophyte (Equisetum arvense), 1 bryophyte (Marchantia polymorpha), and chlorophyte, the alga (Hydrodictyon reticulatum). Thus, BRs are widely distributed in the plant kingdom, including higher and lower plants [7].

On the other hand, a study of the miscellaneous pathways of BRs metabolism in plants reported the existence of around 19 conjugated metabolites in positions C-2, C-3, C-23, C-25, or C-26 [8]. Eight out of nineteen correspond to conjugates formed by esterification in C-3 [9,10,11,12,13,14]. The other eleven conjugated metabolites are formed by glycosylation at C-2, C-3, C-23, C-25, or C-26 [8,13,14,15,16,17,18,19,20]. Some examples of these structures are shown in Figure 1. It seems that conjugated compounds are used by plants to store inactive BRs that can be converted to active forms by de-conjugation reactions. Additionally, the natural conjugates **3**, **4** (Figure 1) were synthesized from 24-epibrassinolide (**2**) [21].

**Figure 2:**
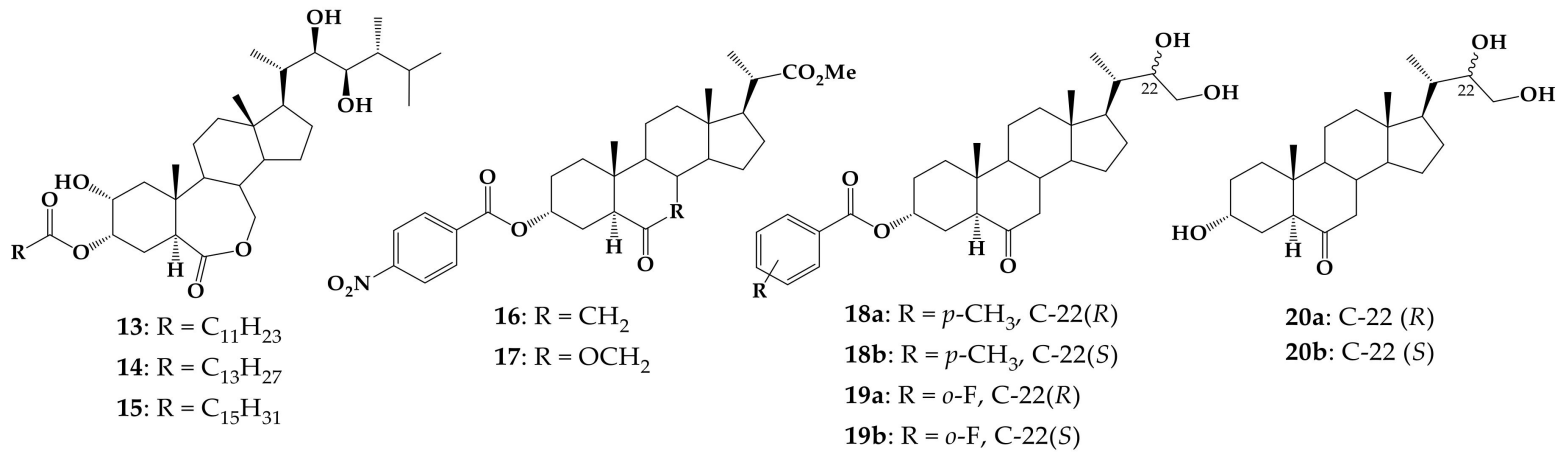
Structure of synthetic brassinosteroids (BRs) analogs conjugated in C-3 (compounds **13**–**19b**) and free synthetic analogs **20a** and **20b**.

On the other hand, a series of C-3 esterified derivatives of 24-epibrassinolide (**13**–**15**) and synthetic BRs analogs (**16**–**19**) (Figure 2) have been reported [21,22].

However, biological evaluations in the Bean Second-Internode Bioassay (BSIB) for compounds **16** and **17** indicated that these analogs are less active than 24-epibrassinolide [22]. These results are in line with previously established structure-activity relationships obtained for natural BRs. These structure-activity relationship (SAR) studies have been made using BSIB and the Rice Lamina Inclination Test (RLIT) [23,24,25], and their main goal is to define general structural requirements for the growth-promoting activity of BRs [24,26,27,28,29]. These results have been used to guide the synthesis of BRs analogs with a variety of structural modifications but keeping those considered essential for biological activity.

Several studies have proved that synthetic BRs analogs with significant structural changes and different substituents, both in the ring and the alkyl chain, can induce similar or even higher biological effects in plants as compared to natural BRs [30,31,32,33,34,35,36]. Some recent reviews of the growth-promoting activity of BRs and their analogs have established novel structural requirements for the existence of biological activity [23,37,38,39]. For example, it has been shown that methyl ethers at C-3 are more active than **1** in the RLIT [40], whereas benzoate esters in the C-3 position were found to be less active than 24-epibrassinolide in the BSIB test [22].

In a previous in silico study, we have assessed the effect on activity of different groups attached to position C-3 of BRs analogs. The results suggest that bulky groups reduce the activity, whereas functionalization with electronegative and hydrophobic groups would increase it [29]. Thus, in this work, we present the synthesis of four new BR 24-norcholane type analogs conjugated with benzoate groups in C-3 (Figure 2, compounds **18a**, **18b**, **19a**, and **19b**). The aromatic ring of the benzoate group contains electron-donor and electron-withdrawing substituents. Their growth-promoting activities were evaluated using RLIT, and the results were compared with those reported for other structurally similar analogs (Figure 2, compounds **20a** and **20b**) [38,41,42].

The synthesis and evaluation of biological activity of these BRs analogs, conjugated in C-3 with benzylic esters, are studied either to get new active molecules or to elucidate if esterification could be a metabolic path for exogenous BRs.

## 2. Results and Discussion

### 2.1. Chemistry

**Scheme 1:**
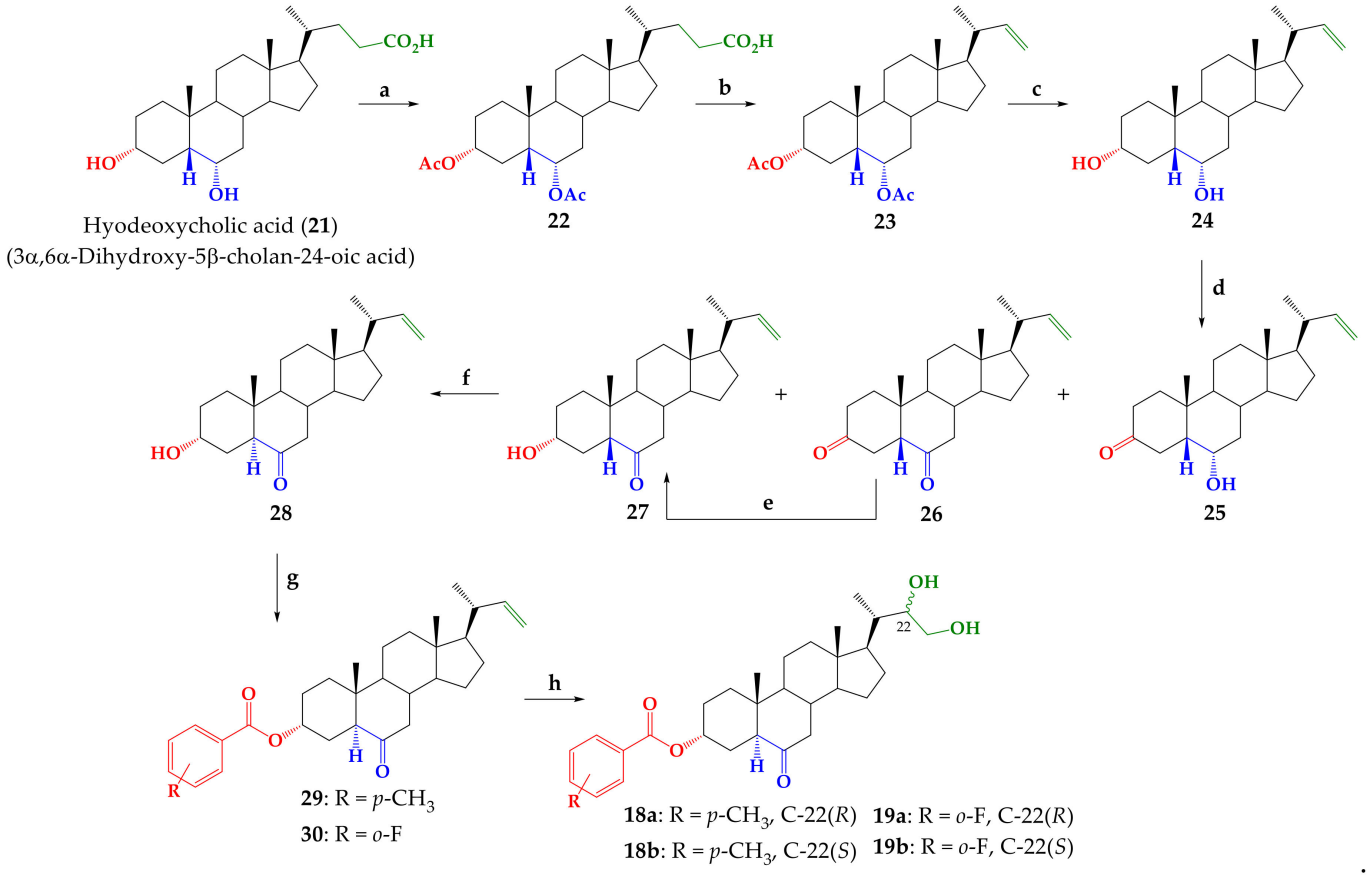
Synthesis of hyodeoxycholic acid derivatives **22**–**30** and C-3 conjugated brassinosteroid analogues **18a**, **18b**, **19a,** and **19b**. *Reagents and conditions*: (**a**) Ac_2_O/DMAP, CH_2_Cl_2_, rt, 48 h, 91.1% yield; (**b**) PhI(OAc)_2_/Cu(OAc)_2_, C_6_H_6_, reflux, 5 h, 99.6% yield; (**c**) K_2_CO_3_ (15% *p*/*v*, H_2_O)/(CH_3_)_2_CO/CH_3_OH, reflux, 7 h, 97.1% yield; (**d**) PCC/CH_2_Cl_2_, rt, 48 h, Column Chromatography (C.C.) separation, **25** (2.4% yield), **26** (19.1% yield), and **27** (40.2% yield); (**e**) NaBH_4_/MeOH, 0–5 °C, 1 h, 76.3% yield; (**f**) HCl/CH_3_OH 2.5% *v*/*v*, rt, 48 h, 74.8% yield; (**g**) *p*-CH_3_C_6_H_4_COCl or *o*-FC_6_H_4_COCl/DMAP/CH_2_Cl_2_, rt, 2 h, **29** (87.9% yield), 3 h, **30** (56.0% yield); (**h**) Dihydroquinidine-Chlorobenzoate(DHQD-CLB)/CH_3_SO_2_NH_2_, K_2_CO_3_/K_3_[Fe(CN)_6_], OsO_4_/(CH_3_)_3_COH/H_2_O, rt, 5 h, **18a**/**18b** (1.0:1.0), 91.6% yield; **19a**/**19b** (1.0:1.0), 80.8% yield.

To obtain the new BR analogs conjugated in C-3 (**18a, 18b, 19a,** and **19b**, Figure 2), the synthetic strategy shown in Scheme 1 was developed. The synthesis of the key intermediate alkene **28** has been previously reported [43], but herein, we have introduced some modifications in the synthesis steps to increase the yields of reactions. In addition, more clear spectroscopic evidence (^1^H- and ^13^C-NMR) is provided [43,44,45].

The standard acetylation (Ac_2_O/*N,N*-dimethylaminopyridine(DMAP)/CH_2_Cl_2_) of hyodeoxycholic acid (**21**) leads to known diacetylated derivative (**22**) in 91.1% yield (ref. 80% yield, [44,45]). In the ^1^H-NMR spectrum of compound **22** (Figure S1, Supplementary Materials), the protons of both acetate groups appear at δ_H_ = 2.02 ppm (3H, s, CH_3_CO_2_-C6) and 1.99 ppm (3H, s, CH_3_CO_2_-C3) [44,45]. While in the ^13^C-NMR spectrum (Figure S1, Supplementary Materials), the observed signals at δ_C_ = 170.56 ppm (CH_3_CO_2_-C6), 170.52 ppm (CH_3_CO_2_-C3), 21.36 ppm (CH_3_CO_2_-C6), and 21.32 ppm (CH_3_CO_2_-C3) confirm the presence of both acetate groups.

Oxidative decarboxylation of the side chain of compound **22**, with the PhI(OAc)_2_/Cu(OAc)_2_ system [44,45], leads to olefin **23** in 99.6% yield (yield data were not reported by other authors). In the ^1^H-NMR of compound **23** (Figure S2, Supplementary Materials), the protons H-22, H*_trans_*-23, and H*_cis_*-23 appear at δ_H_ = 5.65 ppm (ddd, *J* = 17.1, 10.2 and 8.4 Hz), 4.90 ppm (dd, *J* = 17.1 and 2.0 Hz), and 4.81 ppm (dd, *J* = 10.2 and 2.0 Hz), respectively [44,45]. Meanwhile, in the ^13^C-NMR (Figure S2, Supplementary Materials), the carbons C-22 and C-23 appear at δ_C_ = 145.06 and 111.69 ppm, respectively. These signals confirm the presence of terminal alkene.

The saponification of diacetate **23** with the system K_2_CO_3_/acetone/methanol/reflux leads to diol **24** in 97.1% yield (ref. 98% yield, [43]). Although compound **24** was previously reported, no NMR spectroscopic data were reported [43,45]. So, the observed signals in the ^1^H-NMR spectrum (Figure S3, Supplementary Materials) at δ_H_ = 4.02–3.96 ppm (1H, m) and 3.48–3.42 ppm (1H, m) were assigned to carbinolic hydrogens H-6 and H-3, respectively (Table 1). While in the ^13^C-NMR (Figure S3, Supplementary Materials), the carbons C-6 and C-3 appear at δ_C_ = 67.63 and 71.72 ppm, respectively (Table 1). The assignments for the H-6 and H-3 signals were confirmed by the 2D HSQC spectrum of compound **24**.

The subsequent oxidation of compound **24** with the PCC/CH_2_Cl_2_ system produces a mixture of three oxidation products (Scheme 1), which were efficiently separated by flash chromatographic column. So, the least polar product was identified as diketone **26** (19.1% yield), a product of intermediate polarity identified as monoketone **25** (2.4% yield), and the most polar product identified as the desired monoketone **27** (40.2% yield). Diketone **26** was previously obtained by the oxidation of glycol with Jones reagent in 95% yield [45]. Meanwhile, diketone **26** and monoketone **27** were obtained by oxidation with the PDC/CH_2_Cl_2_ system, with 21% and 61.7% yields, respectively [43]. The IR and ^1^H-NMR spectroscopic data for compounds **26** and **27** were consistent with those reported (Figures S5 and S6, Supplementary Materials) [43,45]. However, none of these previous works reported obtaining monoketone **25**. In the ^1^H-NMR spectrum of this compound (Figure S4, Supplementary Materials), the observed signal at δ_H_ = 4.15–4.10 ppm (1H, m) was assigned to carbinolic hydrogen H-6, whereas in the ^13^C-NMR spectrum (Figure S4, Supplementary Materials), the observed signal at δ_C_ = 67.73 ppm corresponds to C-6.

Table 1 shows the differences detected for the main signals observed in the ^1^H- and ^13^C-NMR spectra of compounds **24** to **27**. All this information was confirmed by the 2D HSQC correlation spectra of compounds **25**–**27**.

Diketone **26** was conveniently converted to the desired monoketone **27** by selective reduction with NaBH_4_/MeOH [43] at low temperature (0–5 °C) with 76.3% yield (step e, Scheme 1). The spectroscopic data of this compound and **27**, which was obtained by direct oxidation from **24** (step d, Scheme 1), were identical.

Then, compound **27** was easily isomerized under acid condition (2.5% *v/v* HCl/MeOH) to give the derivative **28** possessing 5α-cholestan-6-one skeleton (74.8% yield) [43,44,46,47,48,49]. The IR, ^1^H- and ^13^C-NMR spectroscopic data registered for compound **28** were consistent with those reported (Figure S7, Supplementary Materials) [43,46].

**Figure 3:**
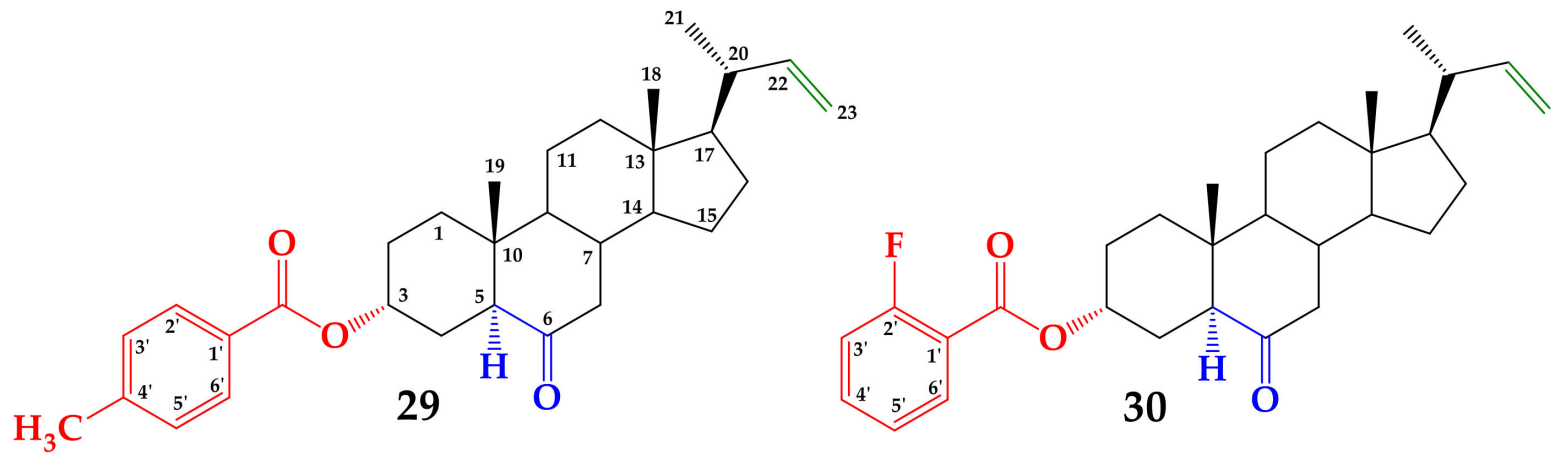
Structures of derivatives **29** and **30** and numbering of aromatic carbon atoms used in this study.

The C-3 benzoylation reactions of **28** were carried out according to the methodology reported for other steroidal nuclei [41,50,51]. So, treatment of **28** with 4-methylbenzoyl chloride/DMAP in CH_2_Cl_2_ and pyridine led to 4-metylbenzoate derivative **29** with 87.9%. Similarly, the reaction of **28** with 2-fluorobenzoyl chloride led to 2-fluorobenzoate derivative **30** with 56.0%. The structures of both derivatives were mainly characterized by ^1^H and ^13^C spectroscopy. For derivative **29**, the presence of aromatic signals at δ_H_ = 8.00 ppm (2H, d, *J* = 9.0 Hz) and 6.93 ppm (2H, d, *J* = 9.0 Hz) were assigned to the hydrogens HAr-2’ and HAr-3′, respectively, whereas the signals appearing at δ_C_ = 163.36, 131.58, 123.35, and 113.70 ppm were assigned to the aromatic carbons C4′, C2′/C6′, C1′, and C3′/C5′ (Figure S8, Supplementary Materials). For derivative **30**, the presence of the aromatic signals at δ_H_ = 7.92 ppm (1H, td, *J* = 7.6 and 1.8 Hz); 7.54–7.48 ppm (1H, m); 7.21 ppm (1H, td, *J* = 7.6 and 1.2 Hz), and 7.13 ppm (1H, ddd, *J* = 10.7, 7.6 and 0.9 Hz) were assigned to the hydrogens HAr-6′, HAr-4′, HAr-3′, and HAr-5′, respectively (Figure S9, Supplementary Materials). In the ^13^C-NMR spectrum (Figure S9, Supplementary Materials), the observed signals at δ_C_ = 161.92 ppm (d, ^1^*J*_CF_ = 259.1 Hz); 134.44 ppm (d, ^3^*J*_CF_ = 8.4 Hz); 132.32 ppm (d, ^3^*J*_CF_ = 0.9 Hz); 124.11 ppm (d, ^4^*J*_CF_ = 3.6 Hz); 119.38 (d, ^2^*J*_CF_ = 9.6 Hz); and 117.05 (d, ^2^*J*_CF_ = 21.6 Hz) were assigned to the aromatic carbons C2′, C4′, C6′, C5′, C1′, and C3′, respectively (Figure 3).

Recently, the synthesis of glycols C22/C23 in steroids with the shortest side chain of 24-nor-5α-cholane type by a Sharpless dihydroxylation reaction has been reported [42]. The results showed that this type of hydroxylation leads to a mixture of C-22 glycols (*R*/*S*) with an approximate 1:1 ratio of both diastereomers [42]. Thus, both olefins **29** and **30** were dihydroxylated following this method and using dihydroquinidine *p*-chlorobenzoate (DHQD-CLB) as a chiral ligand (Scheme 1) [32,42]. The Sharpless dihydroxylation of derivative **29** produced the **18a/18b** diastereoisomer mixture with a total 91.6% yield. The diastereomeric ratio of each glycol in the mixture can be established by the integration of ^1^H-NMR signals assigned to the C-21 methyl group, which appear at δ_H_ = 0.921 and 0.953 ppm in **18a** and **18b** diastereoisomers, respectively. Based on these NMR measurements, the relative ratio of **18a**:**18b** was determined as 1.0:1.0. Subsequently, the diastereoisomers mixture was separated by a semi-preparative HPLC system, allowing obtaining the analogs **18a** and **18b**.

The structure and stereochemistry at C-22 of compounds **18a** and **18b** was established by a simple comparison of ^1^H- and ^13^C-NMR spectra obtained for derivatives **20a** and **20b**, which were previously reported [41,42]. These comparisons considered chemical shifts (δ), coupling constants (*J*), and multiplicities of signals corresponding to H-22, H-23a, H-23b, and CH_3_-21 (^1^H-NMR) and chemical shifts (δ) in ^13^C-NMR of both epimers. The main differences in these spectroscopic parameters are listed in Table 2.

Similarly, a Sharpless dihydroxylation of derivative **30** produced the **19a/19b** diastereoisomers mixture with a total 80.8% yield. The diastereomeric ratio of each glycol in the mixture was 1.0:1.0 (established by the integration of ^1^H-NMR signals assigned to the C-21 methyl group, which appear at δ_H_ = 0.917 and 0.954 ppm in **19a** and **19b** diastereoisomers, respectively). The diastereoisomers mixture was separated by semi-preparative HPLC system, allowing obtaining analogs **19a** and **19b**. Similar to the above, the main differences in spectroscopic parameters of epimers are listed in Table 3.

In summary, four new BRs 24-norcholane type analogs conjugated at the C-3 position with benzoate groups substituted with electron donor and electron-withdrawing groups in the *p*-position (compounds **18a**, **18b**, **19a** and **19b**) have been synthesized and characterized.

### 2.2. Biological

In this work, the activity of new BR 24-norcholane type analogs conjugated at the C-3 position was evaluated using the Rice Lamina Inclination Test. The results of this test were compared with those obtained for other free analogs of 24-norcholane type (analogs **20a** and **20b** [38]) and with brassinolide. This assay was used because of its specificity and high sensitivity for **1** and their analogs [31,52,53]. The bending angles were measured as the difference between the induced angle produced by treatment with each compound and that found for the negative control. Results obtained for **1**, which was used as positive control, and BR analogs **18a, 18b, 19a, 19b, 20a,** and **20b** are listed in Table 4.

**Figure 4:**
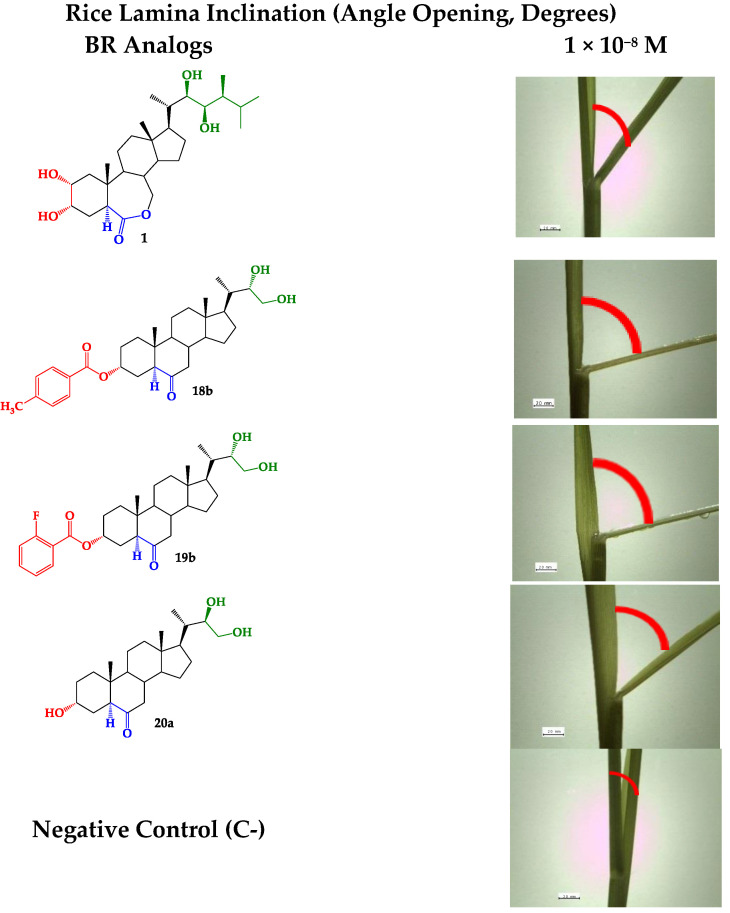
Rice-lamina assays using the second leaf lamina joints of excised leaf segments treated with BR analogs (**18b**, **19b**, and **20a**) at 1 × 10^−8^ M. Brassinolide (**1**) was used as positive control at the same concentrations. The negative control only contained sterile distilled water.

Interestingly, these data clearly indicate that 24-nor-5α-cholane type analogs conjugated at C-3 exhibit interesting growth-promoting activity. These results are in line with previous studies for other analogs of 24-norcholane type [38,48]. All C-3 conjugated analogs exhibit higher activity than brassinolide at the lowest concentrations (1 × 10^−8^ and 1 × 10^−7^ M) (Figure 4 and Table 4).

To simplify the data analysis, we will consider the data obtained at 1 × 10^−8^ and 1 × 10^−7^ M to analyze the correlation between chemical structure and biological activity. The results indicate that at these concentrations, **18b** and **19b** are the most active in the series of conjugated analogs (**19b** was the most active at the concentration of 1 × 10^−8^ M, whereas **18a** was the most active at the concentration of 1 × 10^−7^ M) (see Table 3), and they were more active than the free analogs **20a** and **20b**. Another important effect to consider is related to the configuration on the C-22 carbon of the side chain. Thus, at the concentration of 1 × 10^−8^, analogs **18a** and **19a** with C-22(*R*) configuration are less active than analogs **18b** and **19b** with C-22(*S*) configuration. However, an opposite effect is observed for analogs **20a** and **20b**. Similarly, at the concentration of 1 × 10^−7^ M, the analogs **19b** and **20b** with C-22(*S*) configuration are more active that analogs **19a** and **20a** with C-22(*R*) configuration. However, an opposite effect is observed for analogs **18a** and **18b**. The results observed for the pairs **18a/18b** (at 1 × 10^−7^ M) and **20a/20b** (at 1 × 10^−8^ M) would be aligned with those reported for natural occurring BRs with an intact side chain, which indicates that glycol function with C-22(*R*) and C-23(*R*) configuration appears essential for a high biological activity and are more active than those with C-22(*S*) and C-23(*S*) configuration [3,54]. However, these apparently contradictory structural effects of BRs analogs could be explained in attributed to the shorter side chains. This structural feature could give a greater rotational freedom degree.

## 3. Materials and Methods

### 3.1. Chemistry

All reagents were purchased from commercial suppliers and used without further purification. Melting points were measured on a SMP3 apparatus (Stuart-Scientific, now Merck KGaA, Darmstadt, Germany) and are uncorrected. ^1^H-, ^13^C-, ^13^C-DEPT-135, gs 2D HSQC, and gs 2D HMBC NMR spectra were recorded in CDCl_3_ and MeOD solutions, and they are referenced to the residual peaks of CHCl_3_ at δ = 7.26 ppm and δ = 77.00 ppm for ^1^H and ^13^C, respectively and CD_3_OD at δ = 3.30 ppm and δ = 49.00 ppm for ^1^H and ^13^C, on an Avance 400 Digital NMR spectrometer (Bruker, Rheinstetten, Germany) operating at 400.1 MHz for ^1^H and 100.6 MHz for ^13^C, and JEOL JNM-ECA 500 NMR spectrometer (JEOL, Tokyo, Japan) operating at 500.16 MHz for ^1^H, 125.77 MHz for ^13^C, and 470.62 MHz for ^19^F. Chemical shifts are reported in ppm and coupling constants (*J*) are given in Hz; multiplicities are reported as follows: singlet (s), doublet (d), doublet of doublets (dd), doublet of triplets (dt), triplet (t), quartet (q), multiplet (m), and broad singlet (bs). IR spectra were recorded as KBr disks in a Fourier Transform Infrared (FT-IR) 6700 spectrometer (Nicolet, Thermo Scientific, San Jose, CA, USA) and frequencies are reported in cm^−1^. High-resolution mass spectra (HRMS) were recorded in an API HRMS instrument, and the samples were dissolved in chloroform (or chloroform: methanol; 1:1; *v*/*v*, in the case of hydroxylated compounds) to a concentration of 10 μg mL^−1^. The ASAP (Atmospheric Solids Analysis Probe) was dipped into the sample solution, placed into the ion source, and analyzed in full scan mode. The source of the Synapt G2-Si mass spectrometer (Waters, Manchester, UK) was operated in positive ionization mode (ASAP^+^), if not stated otherwise, at a source temperature of 120 °C. The corona needle current was kept at 5 μA and the collision energy was kept at 4 V. The probe temperature was ramped up from 50 to 600 °C in 3 min. Data were acquired from 50 to 1000 Da with 1.0 s scan time in high-resolution mode. The data were processed using the Masslynx 4.1 software (Waters, Milford, MA, USA). A mass accuracy of 1 ppm or less was achieved with the described instrumentation for all compounds. For analytical TLC, silica gel 60 in a 0.25 mm layer was used, and TLC spots were detected by heating after spraying with 10% H_2_SO_4_ in H_2_O. Chromatographic separations were carried out by conventional column on silica gel 60 (230–400 mesh) using EtOAc–hexane gradients of increasing polarity. All organic extracts were dried over anhydrous magnesium sulfate and evaporated under reduced pressure, below 40 °C. The HPLC system consisted of a Waters semi-preparative HPLC system including a quaternary pump, a liquid handler, and UV-Vis and Evaporative Light Scattering Detector (ELSD) detectors. The semi-preparative column was filled with silica gel.

#### 3.1.1. Synthesis

##### 3α,6α-Diacetoxy-5β-cholan-24-oic acid (**22**)

To a solution of hyodeoxycholic acid (**21**) (25.4 g, 64.62 mmol) in 400 mL of CH_2_Cl_2_ (DCM), 150 mg of DMAP, 2 mL of pyridine, and 24.4 mL (257.6 mmol) of Ac_2_O were added. The reaction mixture was kept under constant stirring and room temperature for 48 h. The end of the reaction was verified by TLC; then, the mixture was concentrated to a volume approximately 50 mL under reduced pressure. Then, EtOAc (200 mL) and 200 mL of HCl solution (1 × 10^−5^ M) were added. The organic layer was separated and washed with water (4 × 50 mL), with saline (NaCl) solution (3 × 50 mL) until pH = 5, dried over Na_2_SO_4_, and filtered. The solvent was evaporated under reduced pressure, and the crude was re-dissolved in CH_2_Cl_2_ (16 mL) and chromatographed on silica gel with EtOAc/hexane (20%, 200 mL). Compound **22** (28.06 g 91.1% yield) was a colorless solid, m.p. = 109.7-110.9 °C (106–110 °C [44]). IR ν_max_ (cm^−1^): 3527 (O-H); 2948; 2899 and 2870 (C-H); 1738 (C=O); 1722 (C=O); 1681 (C=O); 1455 (CH_2_); 1364 (CH_3_); 1256 (C-O); 1242(C-O); 1027 (C-O). ^1^H-NMR (400.1 MHz, CDCl_3_) (Figure S1, Supplementary Materials): δ (ppm) = 5.14–5.10 (1H, m, H-6); 4.71–4.65 (1H, m, H-3); 2.37 (1H, ddd, *J* = 15.3, 10.1 and 5.0 Hz, H-23a); 2.23 (1H, ddd, *J* = 16.0, 10.1, and 6.4 Hz, H-23b); 2.02 (3H, s, CH_3_CO_2_-C6); 1.99 (3H, s, CH_3_CO_2_-C3); 1.97–1.94 (1H, m, H-5); 0.95 (3H, s, H-19); 0.90 (3H, d, *J* = 6.4 Hz, H-21); 0.62 (3H, s, H-18). ^13^C-NMR (100.6 MHz, CDCl_3_) (Figure S1, Supplementary Materials) δ (ppm) = 180.09 (C-24); 170.56 (CH_3_CO_2_-C6); 170.52 (CH_3_CO_2_-C3); 73.66 (C-3); 70.92 (C-6); 56.06 (C-14); 55.84 (C-17); 45.28 (C-9); 42.81 (C-13); 39.78 (C-5 and C-12); 35.97 (C-10); 35.18 (C-20); 34.96 (C-7); 34.54 (C-8); 31.20 (C-1); 30.93 (C-22); 30.64 (C-23); 28.01 (C-4); 26.35 (C-16); 26.15 (C-2); 24.00 (C-15); 23.19 (C-19); 21.36 (CH_3_CO_2_-C6); 21.32 (CH_3_CO_2_-C3); 20.61 (C-11); 18.17 (C-21); 11.95 (C-18).

##### 24-Nor-5β-cholan-22-ene-3α,6α-diyl diacetate (**23**)

To a solution of **22** (2.00 g, 4.20 mmol) in dry benzene (150 mL) were added Cu(OAc)_2_*H_2_O (200 mg, 1.0 mmol) and pyridine (2.5 mL). Then, under reflux, PhI(OAc)_2_ (7.04 g, 21.5 mmol) was added in four portions at hourly intervals. After the addition was completed, the reaction was continued for 1 h. The end of the reaction was verified by TLC, and then, the mixture was filtered, and the solvent was evaporated under reduced pressure. The crude was re-dissolved in DCM (5 mL) and chromatographed on silica gel with PE/EtOAc mixtures of increasing polarity (19.8:0.2 -> 15.8:4.2). The reaction was repeated 5 times under identical conditions. Compound **23** (9.0 g, 99.6% yield) was obtained as a colorless solid, m.p. = 89.0–90.9 °C (88–89 °C [45]). IR ν_max_ (cm^−1^): 3082 (CH=CH_2_); 2940; 2887 and 2867 (C-H); 1740 (C=O); 1727 (C=O); 1633 (C=C); 1460 (CH_2_); 1366 (CH_3_); 1244 (C-O); 1026 (C-O); 908 (CH=CH_2_). ^1^H-NMR (400.1 MHz, CDCl_3_) (Figure S2, Supplementary Materials): δ (ppm) = 5.65 (1H, ddd, *J* = 17.1, 10.2, and 8.4 Hz, H-22); 5.16–5.13 (1H, m, H-6); 4.90 (1H, dd, *J* = 17.1 and 2.0 Hz, H-23a); 4.81 (1H, dd, *J* = 10.2 and 2.0 Hz, H-23b); 4.71–4.69 (1H, m, H-3); 2.07–2.03 (1H, m, H-20); 2.04 (3H, s, CH_3_CO_2_-C6); 2.01 (3H, s, CH_3_CO_2_-C3); 2.00–1.94 (1H, m, H-5); 1.02 (3H, d, *J* = 6.6 Hz, H-21); 0.97 (3H, s, H-19); 0.67 (3H, s, H-18). ^13^C-NMR (100.6 MHz, CDCl_3_) (Figure S2, Supplementary Materials) δ (ppm) = 170.47 (CH_3_CO_2_-C6); 170.45 (CH_3_CO_2_-C3); 145.06 (C-22); 111.69 (C-23); 73.70 (C-3); 70.95 (C-6); 56.21 (C-14); 55.59 (C-17); 45.39 (C-9); 42.83 (C-13); 41.13 (C-20); 39.93 (C-5); 39.79 (C-12); 36.07 (C-10); 35.06 (C-1); 34.63 (C-8); 31.30 (C-7); 28.36 (C-16); 26.44 (C-2); 26.25 (C-4); 24.09 (C-15); 23.27 (C-19); 21.41 (CH_3_CO_2_-C6); 21.37 (CH_3_CO_2_-C3); 20.68 (C-11); 20.07 (C-21); 12.18 (C-18).

##### 24-Nor-5β-chol-22-ene-3α,6α-diol (**24**)

To a solution of **23** (8.06 g, 18.71 mmol) in a mixture 1:1 of acetone/MeOH (60 mL), a 15% aqueous solution of K_2_CO_3_ (37.4 mmol) was added. The suspension was stirred and refluxed for 7 h. The end of the reaction was verified by TLC. Then, the solvent was removed, the residue was diluted with EtOAc (80 mL), and the mixture was washed with 3 × 80 mL of HCl solution (1 ×10^−3^ M). The organic layer was dried over Na_2_SO_4_ and filtered. The solvent was evaporated under reduced pressure, and the crude was re-dissolved in CH_2_Cl_2_ (15 mL) and chromatographed on silica gel with PE/EtOAc mixtures of increasing polarity (19.8:0.2 -> 7.2:12.8). Compound **24** (6.3 g; 97.1% yield) was obtained as a colorless solid, m.p. = 154.4–155.2 °C (150–152 °C [45]). IR ν_max_ (cm^−1^): 3381 (O-H); 3088 (CH=CH_2_); 2934, 2889, and 2868 (C-H); 1637 (C=C); 1462 (CH_2_); 1336 (CH_3_); 1269 (C-O); 1043 (C-O); 912 (CH=CH_2_). ^1^H-NMR (400.1 MHz, Acetone) (Figure S3, Supplementary Materials): δ (ppm) = 5.66 (1H, ddd, *J* = 17.4, 10.0, and 8.6 Hz, H-22); 4.90 (1H, dd, *J* = 17.1 and 2.0 Hz, H-23a); 4.82 (1H, dd, *J* = 10.2 and 2.0 Hz, H-23b); 4.02–3.96 (1H, m, H-6); 3.48–3.42 (1H, m, H-3); 1.02 (3H, d, *J* = 6.6 Hz, H-21); 0.91 (3H, s, H-19); 0.66 (3H, s, H-18). ^13^C-NMR (100.6 MHz, Acetone) (Figure S3, Supplementary Materials): δ (ppm) = 146.00 (C-22); 112.05 (C-23); 71.72 (C-3); 67.63 (C-6); 57.20 (C-14); 56.40 (C-17); 49.70 (C-9); 43.51 (C-13); 42.09 (C-20); 40.89 (C-5); 40.80 (C-12); 36.65 (C-1); 36.55 (C-10); 35.97 (C-7); 35.70 (C-8); 31.43 (C-4); 30.05 (C-2); 29.15 (C-16); 24.90 (C-15); 24.10 (C-19); 21.54 (C-11); 20.55 (C-21); 12.55 (C-18).

##### 6α-Hydroxy-24-nor-5β-chol-22-en-3-one (**25**), 24-nor-5β-chol-22-ene-3,6-dione (**26**) and 3α-hydroxy-24-nor-5β-chol-22-en-6-one (**27**)

A solution of **24** (6.0 g, 17.3 mmol) in DCM (100 mL) with 3.76 g (17.3 mmol) of Pyridinium chlorochromate (PCC) in 60 mL of DCM (added by slow drip) was slowly stirred for 48 h at room temperature. The end of the reaction was verified by TLC; then, the reaction mixture was filtered on alumina and washed with ethyl acetate (20 mL). The solvent was evaporated under reduced pressure, and the crude was re-dissolved in CH_2_Cl_2_ (10 mL) and chromatographed on silica gel with PE/EtOAc mixtures of increasing polarity (19.8:0.2 -> 8.8:11.2). Four fractions were obtained: Fraction I, 1.14 g (19.1% yield) of compound **26**; Fraction II, 0.141 g (2.4% yield) of compound **25**; Fraction III, 2.39 g (40.2% yield) of compound **27;** and Fraction IV, 3.58 g of unreacted **24**. The reaction was repeated with compound **24** twice with another 5.5 g each and **26** (2.08 g), **25** (4.28 g), and **27** (0.258 g) were obtained.

Compound **25** was obtained as a colorless solid. ^1^H-NMR (400.1 MHz, CDCl_3_) (Figure S4, Supplementary Materials): δ (ppm) = 5.68 (1H, ddd, *J* = 17.1, 10.2, and 8.4 Hz, H-22); 4.93 (1H, dd, *J* = 17.1 and 2.0 Hz, H-23a); 4.84 (1H, dd, *J* = 10.2 and 2.0 Hz, H-23b); 4.15–4.10 (1H, m, H-6); 2.42–2.39 (2H, m, H-4); 1.05 (3H, d, *J* = 6.6 Hz, H-21); 1.03 (3H, s, H-19); 0.72 (3H, s, H-18). ^13^C-NMR (100.6 MHz, CDCl_3_) (Figure S4, Supplementary Materials): δ (ppm) = 212.63 (C-3); 145.00 (C-22); 111.77 (C-23); 67.73 (C-6); 56.17 (C-14); 55.55 (C-17); 50.16 (C-9); 42.79 (C-13); 41.15 (C-20); 40.31 (C-5); 39.74 (C-12); 37.08 (C-1); 37.06 (C-7); 36.24 (C-10); 36.02 (C-4); 34.56 (C-8); 34.40 (C-2); 28.35 (C-16); 24.17 (C-15); 22.85 (C-19); 21.08 (C-11); 20.10 (C-21); 12.23 (C-18).

Compound **26** was obtained as a colorless solid, m.p. = 177.5–178.9 °C (197–200 °C [45]). IR ν_max_ (cm^−1^): 3073 (CH=CH_2_); 2964, 2947, 2873, and 2855 (C-H); 1716 (C=O); 1693 (C=O); 1632 (C=C); 1466 (CH_2_); 1382 (CH_3_); 1245 (C-O); 1216 (C-O); 908 (CH=CH_2_). ^1^H-NMR (400.1 MHz, CDCl_3_) (Figure S5, Supplementary Materials): δ (ppm) = 5.68 (1H, ddd, *J* = 17.1, 10.2, and 8.4 Hz, H-22); 4.94 (1H, dd, *J* = 17.1 and 2.0 Hz, H-23a); 4.86 (1H, dd, *J* = 10.2 and 2.0 Hz, H-23b); 2.67 (1H, dd, *J* = 15.0 and 13.2 Hz, H-4a); 2.50 (1H, dd, *J* = 12.5 and 4.9 Hz, H-5); 2.42 (1H, dd, *J* = 14.3 and 5.3 Hz, H-1a); 2.36 (1H, dd, *J* = 5.2 and 1.9 Hz, H-4b); 1.07 (3H, d, *J* = 6.5 Hz, H-21); 0.98 (3H, s, H-19); 0.74 (3H, s, H-18). ^13^C-NMR (100.6 MHz, CDCl_3_) (Figure S5, Supplementary Materials): δ (ppm) = 210.82 (C-6); 208.65 (C-3); 144.72 (C-22); 111.99 (C-23); 59.74 (C-5); 56.86 (C-14); 55.40 (C-17); 43.02 (C-13); 42.16 (C-7); 41.06 (C-20); 40.98 (C-9); 39.90 (C-4); 39.41 (C-12); 38.31 (C-10); 36.69 (C-8); 36.49 (C-1); 35.78 (C-2); 28.21 (C-16); 23.93 (C-15); 22.48 (C-19); 21.30 (C-11); 20.09 (C-21); 12.17 (C-18).

Compound **27** was obtained as a colorless solid, m.p. = 151.9-153.6 °C. IR ν_max_ (cm^−1^): 3288 (O-H); 3074 (CH=CH_2_); 2970, 2949, and 2867 (C-H); 1702 (C=O); 1637 (C=C); 1458 (CH_2_); 1379 (CH_3_); 1248 (C-O); 1064 (C-O); 912 (CH=CH_2_). ^1^H-NMR (400.1 MHz, CDCl_3_) (Figure S6, Supplementary Materials): δ (ppm) = 5.67 (1H, ddd, *J* = 17.1, 10.1, and 8.4 Hz, H-22); 4.93 (1H, dd, *J* = 17.1 and 2.0 Hz, H-23a); 4.85 (1H, dd, *J* = 10.1 and 2.0 Hz, H-23b); 3.70–3.62 (1H, m, H-3); 2.20–2.18 (2H, m, H-7); 2.14 (1H, dd, *J* = 12.1 and 5.1 Hz, H-5); 1.05 (3H, d, *J* = 6.6 Hz, H-21); 0.86 (3H, s, H-19); 0.70 (3H, s, H-18). ^13^C-NMR (100.6 MHz, CDCl_3_) (Figure S6, Supplementary Materials): δ (ppm) = 213.89 (C-6); 144.89 (C-22); 111.87 (C-23); 70.18 (C-3); 59.41 (C-5); 56.89 (C-14); 55.41 (C-17); 43.04 (C-13); 42.93 (C-7); 41.11 (C-20); 40.07 (C-9); 39.53 (C-12); 37.99 (C-10); 37.07 (C-8); 34.86 (C-1); 34.38 (C-4); 29.86 (C-2); 28.26 (C-16); 23.99 (C-15); 23.17 (C-19); 20.84 (C-11); 20.08 (C-21); 12.15 (C-18).

##### 3α-Hydroxy-24-nor-5β-chol-22-en-6-one (27) from 24-nor-5β-chol-22-ene-3,6-dione (**26**)

A solution of compound **26** (3.22 g, 9.4 mmol) was prepared in 100 mL of 1:1 MeOH/THF mixture. This solution was placed in a bath of ice-water between 0 and 5 °C. Subsequently, 117.9 mg (3.15 mmol) of NaBH_4_ were added in four portions (approximately 29.5 mg each) maintaining the temperature and with slow stirring. The end of the reaction was verified by TLC, 10 mL of acetone, and subsequently, 5 mL of HCl 2.5% were added, maintaining the reaction temperature. The reaction mixture was concentrated by evaporation under reduced pressure to a volume of about 15 mL, and then, EtOAc (50 mL) was added. The organic layer was washed with saturated solution of NaHCO_3_ (20 mL) and water (2 × 30 mL), dried over anhydrous MgSO_4_, and filtered. The solvent was evaporated under reduced pressure. The crude was re-dissolved in CH_2_Cl_2_ (5 mL) and chromatographed on silica gel with PE/EtOAc mixtures of increasing polarity (9.8:02 -> 5.8:4.2). Two fractions were obtained: Fraction I, 0.753 g of unreacted compound **26**; Fraction II, 2.47 g (76.3% yield) of compound **27**. The melting point and spectroscopic properties (^1^H- and ^13^C-NMR) of compounds **26** and **27** were identical to those reported above for the direct oxidation of compound **24**.

##### 3α-Hydroxy-24-nor-5α-chol-22-en-6-one (**28**)

Compound **27** (6.08 g, 17.65 mmol) was dissolved in 100 mL of 2.5% *v/v* HCl-MeOH at room temperature and constant agitation for 48 h. The end of the reaction was verified by TLC. The solvent was evaporated under reduced pressure, and the crude was re-dissolved in 60 mL of EtOAc. The organic layer was washed with saturated solution of NaHCO_3_ (2 × 15 mL) and water (2 × 30 mL), dried over MgSO_4_, and filtered. The solvent was evaporated under reduced pressure. The crude was re-dissolved in CH_2_Cl_2_ (5 mL) and chromatographed on silica gel with PE/EtOAc mixtures of increasing polarity (9.8:0.2 -> 4.0:6.0). Two fractions were obtained: Fraction I, 4.55 g (74.8% yield) of compound **28** and Fraction II, 1.15 g of unreacted compound **27**. Compound **28** was obtained as a colorless solid, m.p. = 169.9–170.9 °C. ^1^H-NMR (400.1 MHz, CDCl_3_) (Figure S7, Supplementary Materials): δ (ppm) = 5.68 (1H, ddd, *J* = 17.0, 10.1, and 8.3 Hz, H-22); 4.93 (1H, dd, *J* = 17.0 and 2.0 Hz, H-23a); 4.84 (1H, dd, *J* = 10.1 and 2.0 Hz, H-23b); 4.20–4.17 (1H, m, H-3); 2.74 (1H, t, *J* = 7.9 Hz, H-5); 2.32 (1H, dd, *J* = 13.1 and 4.5 Hz, H-7α); 2.15–1.97 (3H, m, H-20, H-12α, and H-7β); 1.06 (3H, d, *J* = 6.6 Hz, H-21); 0.75 (3H, s, H-19); 0.71 (3H, s, H-18). ^13^C-NMR (100.6 MHz, CDCl_3_) (Figure S7, Supplementary Materials): δ (ppm) = 212.67 (C-6); 144.96 (C-22); 111.81 (C-23); 65.47 (C-3); 56.84 (C-14); 55.37 (C-17); 53.88 (C-9); 51.69 (C-5); 46.86 (C-7); 42.96 (C-13); 41.57 (C-10); 41.12 (C-20); 39.41 (C-12); 37.98 (C-8); 31.69 (C-1); 28.19 (C-2 and C-4); 27.71 (C-16); 23.93 (C-15); 21.07 (C-11); 20.07 (C-21); 12.32 (C-19); 12.20 (C-18). HRMS (API+) (Figure S14, Supplementary Materials): *m*/*z* calculated for C_23_H_37_O_2_ ([M + H]^+^) 345.2794; found 345.2795.

##### 6-Oxo-24-nor-5α-chol-22-en-3α-yl 4-methylbenzoate (**29**)

A solution of compound **28** (150 mg, 0.44 mmol) and DMAP (44 mg, 0.36 mmol) was prepared in 3 mL of anhydrous pyridine. To this solution, 4-methylbenzoyl chloride 174 μL (1.32 mmol) was added by slow dripping, and the reaction was maintained at room temperature with constant stirring for 2 h. The end of the reaction was verified by TLC. After completion of the reaction, 3 mL of hot water was added. After an additional 20 min of stirring, the mixture was extracted with EtOAc (20 mL) and washed successively with saturated NaHCO_3_ solution (2 × 10 mL) and water (2 × 10 mL), dried over anhydrous MgSO_4_, and filtered. The solvent was evaporated under reduced pressure, and the crude was re-dissolved in CH_2_Cl_2_ (5 mL) and chromatographed on silica gel with EtOAc/cyclohexane (1.0:19) mixture. Compound **29** (117 mg, 87.9% yield) was obtained as a colorless solid, m.p. = 133.3–135.2 °C. ^1^H-NMR (500.16 MHz, CDCl_3_) (Figure S8, Supplementary Materials): δ (ppm) = 7.90 (2H, d, *J* = 8.0 Hz, HAr-2′); 7.24 (2H, d, *J* = 8.0 Hz, HAr-3′); 5.65 (1H, ddd, *J* = 17.1, 10.1 and 8.6 Hz, H-22); 5.36–5.34 (1H, m, H-3); 4.91 (1H, ddd, *J* = 17.1, 1.8 and 0.9 Hz, H-23a); 4.82 (1H, dd, *J* = 10.1 and 1.8 Hz, H-23b); 2.66 (1H, dd, *J* = 12.5 and 3.1 Hz, H-5); 2.41 (3H, s, CH_3_-Ar); 2.32 (1H, dd, *J* = 13.1 and 4.6 Hz, H-7α); 1.03 (3H, d, *J* = 6.7 Hz, H-21); 0.786 (3H, s, H-19); 0.695 (3H, s, H-18). ^13^C-NMR (125.77 MHz, CDCl_3_) (Figure S8, Supplementary Materials): δ (ppm) = 211.92 (C-6); 165.78 (CO_2_-Ar); 145.01 (C-22); 143.61 (C4′-Ar); 129.60 (C2′-Ar and C6′-Ar); 129.18 (C3′-Ar and C5′-Ar); 128.18 (C1′-Ar); 111.95 (C-23); 69.37 (C-3); 56.86 (C-14); 55.50 (C-17); 54.09 (C-9); 53.01 (C-5); 46.85 (C-7); 43.05 (C-13); 41.48 (C-10); 41.24 (C-20); 39.44 (C-12); 38.06 (C-8); 32.81 (C-1); 28.31 (C-16); 25.57 (C-2); 25.25 (C-4); 24.02 (C-15); 21.79 (CH_3_-Ar); 21.22 (C-11); 20.16 (C-21); 12.57 (C-19); 12.31 (C-18). HRMS (API+) (Figure S15, Supplementary Materials): *m*/*z* calculated for C_31_H_43_O_3_ ([M + H]^+^) 463.3212; found 463.3212.

##### 6-Oxo-24-nor-5α-chol-22-en-3α-yl 2-fluorobenzoate (**30**)

A solution of compound **29** (120 mg, 0.348 mmol) and DMAP (35 mg, 0.286 mmol) was prepared in 3 mL of anhydrous pyridine. To this solution, 2-fluorobenzoyl chloride 123.5 μL (1.05 mmol) was added by slow dripping, and the reaction was maintained at room temperature with constant stirring for 3 h. The end of the reaction was verified by TLC. After completion of the reaction, 3 mL of hot water was added. After an additional 20 min of stirring, the mixture was extracted with EtOAc (20 mL) and washed successively with saturated NaHCO_3_ solution (2 × 10 mL) and water (2 × 10 mL), dried over anhydrous MgSO_4_, and filtered. The solvent was evaporated under reduced pressure, and the crude was re-dissolved in CH_2_Cl_2_ (5 mL) and chromatographed on silica gel with EtOAc/cyclohexane (1.0:19) mixture. Compound **30** (91 mg, 56.0% yield) was obtained as a colorless solid, m.p. = 120.6–120.8 °C. ^1^H-NMR (500.16 MHz, CDCl_3_) (Figure S9, Supplementary Materials): δ (ppm) = 7.92 (1H, td, *J* = 7.6 and 1.8 Hz, HAr-6′); 7.54–7.48 (1H, m, HAr-4′); 7.21 (1H, td, *J* = 7.6 and 1.2 Hz, HAr-3′); 7.13 (1H, ddd, *J* = 10.7, 7.6 and 0.9 Hz, HAr-5′); 5.65 (1H, ddd, *J* = 17.1, 10.1, and 8.6 Hz, H-22); 5.42–5.39 (1H, m, H-3); 4.90 (1H, ddd, *J* = 17.1, 1.8, and 0.9 Hz, H-23a); 4.82 (1H, dd, *J* = 10.1 and 1.8 Hz, H-23b); 2.71 (1H, dd, *J* = 12.5 and 3.1 Hz, H-5); 2.31 (1H, dd, *J* = 13.1 and 4.6 Hz, H-7α); 1.03 (3H, d, *J* = 6.7 Hz, H-21); 0.781 (3H, s, H-19); 0.692 (3H, s, H-18). ^13^C-NMR (125.77 MHz, CDCl_3_) (Figure S9, Supplementary Materials): δ (ppm) = 212.04 (C-6); 163.85 (d, ^3^*J*_CF_ = 3.6 Hz, CO_2_-Ar); 161.92 (d, ^1^*J*_CF_ = 259.1 Hz, C2′-Ar); 145.02 (C-22); 134.44 (d, ^3^*J*_CF_ = 8.4 Hz, C4′-Ar); 132.32 (d, ^3^*J*_CF_ = 0.9 Hz, C6′-Ar); 124.11 (d, ^4^*J*_CF_ = 3.6 Hz, C5′-Ar); 119.38 (d, ^2^*J*_CF_ = 9.6 Hz, C1′-Ar); 117.05 (d, ^2^*J*_CF_ = 21.6 Hz, C3′-Ar); 111.93 (C-23); 70.34 (C-3); 56.82 (C-14); 55.50 (C-17); 53.99 (C-9); 52.79 (C-5); 46.84 (C-7); 43.05 (C-13); 41.42 (C-10); 41.23 (C-20); 39.43 (C-12); 38.06 (C-8); 32.59 (C-1); 28.30 (C-16); 25.51 (C-2); 25.22 (C-4); 24.01 (C-15); 21.21 (C-11); 20.17 (C-21); 12.63 (C-19); 12.30 (C-18). ^19^F-NMR (470.62 MHz, CDCl_3_) δ (ppm) = 108.90 (s, 1F). HRMS (API+) (Figure S16, Supplementary Materials): *m*/*z* calculated for C_30_H_40_O_3_F ([M + H]^+^) 467.2961; found 467.2962.

##### (22*R*)-22,23-Dihydroxy-6-oxo-24-nor-5α-cholan-3α-yl 4-methylbenzoate (**18a**) and (22*S*)-22,23-dihydroxy-6-oxo-24-nor-5α-cholan-3α-yl 4-methylbenzoate (**18b**)

To a mixture of *t*-butanol/water (10 mL, 1:1 *v*/*v*) and alkene **29** (100 mg, 0.22 mmol), DHQD-CLB (20.1 mg; 0.043 mmol), CH_3_SO_2_NH_2_ (41.12 mg; 0.43 mmol), K_2_CO_3_ (179.2 mg; 1.3 mmol), and K_3_[Fe(CN)_6_] (427.0 mg; 1.3 mmol) were added; then, the mixture was homogenized by magnetic stirring for 10 min. Later, 100 μL of OsO_4_ solution (1.0 g, 0.562 mmol in 20 mL of *t*-butanol) were added, and the mixture reaction was stirred at room temperature for 5 h. The end of the reaction was verified by TLC; then, H_2_O (10 mL) and a saturated solution of Na_2_S_2_O_3_^.^5H_2_O (2 mL) were added. The mixture was stirred for another 20 min. Later, it was extracted with EtOAc (2 × 35 mL) and washed with water (2 × 35 mL), and both organic phases were combined, dried over MgSO_4_, and filtered. The solvent was evaporated under reduced pressure. The crude was re-dissolved in CH_2_Cl_2_ (1.0 mL) and chromatographed on silica gel with EtOAc/cyclohexane (16:4) mixture. A mixture of **18a**/**18b** = 1.0/1.0 was obtained (93 mg; 91.6% yield). Separation by HPLC of an analytical sample allowed the separation and obtaining of the pure compounds **18a** and **18b**.

Compound **18a** was obtained as a colorless solid, m.p. = 74.1 ± 4.6 °C. ^1^H-NMR (500.16 MHz, CDCl_3_) (Figure S10, Supplementary Materials): δ (ppm) = 7.90 (2H, d, *J* = 8.3 Hz, HAr-2′); 7.24 (2H, d, *J* = 8.3 Hz, HAr-3′); 5.36–5.34 (1H, m, H-3); 3.80 (1H, ddd, *J* = 8.9, 3.3, and 1.2 Hz, H-23a); 3.66–3.61 (1H, m, H-22); 3.52 (1H, dd, *J* = 10.8 and 3.3 Hz, H-23b); 2.66 (1H, dd, *J* = 12.5 and 3.1 Hz, H-5); 2.41 (3H, s, CH_3_-Ar); 2.32 (1H, dd, *J* = 13.1 and 4.6 Hz, H-7α); 0.921 (3H, d, *J* = 6.7 Hz, H-21); 0.783 (3H, s, H-19); 0.681 (3H, s, H-18). ^13^C-NMR (125.77 MHz, CDCl_3_) (Figure S10, Supplementary Materials): δ (ppm) = 211.91 (C-6); 165.82 (CO_2_-Ar); 143.65 (C4′-Ar); 129.61 (C2′-Ar, C6′-Ar); 129.20 (C3′-Ar, C5′-Ar); 128.15 (C1′-Ar); 74.14 (C-22); 69.36 (C-3); 66.16 (C-23); 56.71 (C-14); 53.96 (C-17); 53.00 (C-9); 52.47 (C-5); 46.79 (C-7); 42.94 (C-13); 41.46 (C-10); 39.53 (C-20); 38.09 (C-12); 37.97 (C-8); 32.78 (C-1); 27.72 (C-16); 25.55 (C-2); 25.24 (C-4); 23.95 (C-15); 21.78 (CH_3_-Ar); 21.22 (C-11); 12.73 (C-21); 12.55 (C-19); 12.00 (C-18). HRMS (API+) (Figure S17, Supplementary Materials): calculated for C_31_H_45_O_5_ ([M + H]^+^) 497.3267, found 497.3262.

Compound **18b** was obtained as a colorless solid, m.p. = 83.0 ± 5.0 °C. ^1^H-NMR (500.16 MHz, CDCl_3_) (Figure S11, Supplementary Materials): δ (ppm) = 7.89 (2H, d, *J* = 8.2 Hz, HAr-2′); 7.23 (2H, d, *J* = 8.2 Hz, HAr-3′); 5.36–5.34 (1H, m, H-3); 3.83–3.76 (1H, m, H-23a); 3.69–3.57 (1H, m, H-23b); 3.51 (1H, t, *J* = 10.2 Hz, H-22); 2.65 (1H, dd, *J* = 12.5 and 3.1 Hz, H-5); 2.41 (3H, s, CH_3_-Ar); 2.32 (1H, dd, *J* = 13.1 and 4.6 Hz, H-7α); 0.953 (3H, d, *J* = 7.0 Hz, H-21); 0.778 (3H, s, H-19); 0.679 (3H, s, H-18). ^13^C-NMR (125.77 MHz, CDCl_3_) (Figure S11, Supplementary Materials): δ (ppm) = 211.82 (C-6); 165.79 (CO_2_-Ar); 143.65 (C4′-Ar); 129.60 (C2′-Ar and C6′-Ar); 129.18 (C3′-Ar and C5′-Ar); 128.16 (C1′-Ar); 73.92 (C-22); 69.36 (C-3); 62.54 (C-23); 56.42 (C-14); 54.06 (C-17); 53.02 (C-9); 52.92 (C-5); 46.79 (C-7); 43.48 (C-13); 41.43 (C-10); 40.15 (C-20); 39.47 (C-12); 38.04 (C-8); 32.81 (C-1); 27.47 (C-16); 25.55 (C-2); 25.23 (C-4); 24.12 (C-15); 21.78 (CH_3_-Ar); 21.21 (C-11); 13.15 (C-21); 12.55 (C-19); 11.85 (C-18). HRMS (API+) (Figure S18, Supplementary Materials): calculated for C_31_H_45_O_5_ ([M + H]^+^) 497.3267, found 497.3265.

##### (22*R*)-22,23-Dihydroxy-6-oxo-24-nor-5α-cholan-3α-yl 2-fluorobenzoate (**19a**) and (22*S*)-22,23-dihydroxy-6-oxo-24-nor-5α-cholan-3α-yl 2-fluorobenzoate (**19b**)

To a mixture of *t*-butanol/water (8 mL; 1:1 *v*/*v*), alkene **29** (75 mg; 0.161 mmol), DHQD-CLB (15.9 mg; 0.0343 mmol), CH_3_SO_2_NH_2_ (32.61 mg; 0.343 mmol), K_2_CO_3_ (142.2 mg; 1.03 mmol), and K_3_[Fe(CN)_6_] (338,7 mg; 1,03 mmol) were added; then, the mixture was homogenized by magnetic stirring for 10 min. Later, 100 μL of OsO_4_ solution (1.0 g, 0.562 mmol in 20 mL of *t*-butanol) were added, and the mixture reaction was stirred at room temperature for 5 h. The end of the reaction was verified by TLC; then, H_2_O (10 mL) and a saturated solution of Na_2_S_2_O_3_^.^5H_2_O (2 mL) were added. The mixture was stirred for another 20 min. Later, it was extracted with EtOAc (2 × 35 mL) and washed with water (2 × 35 mL), and both organic phases were combined, dried over MgSO_4_, and filtered. The solvent was evaporated under reduced pressure. The crude was re-dissolved in CH_2_Cl_2_ (1.0 mL) and chromatographed on silica gel with an EtOAc/cyclohexane (16:4) mixture. A mixture of **19a**/**19b** = 1.0/1.0 was obtained (65 mg: 80.8% yield). The separation by HPLC of an analytical sample allowed the separation and obtaining of the pure compounds **19a** and **19b**.

Compound **19a** was obtained as a colorless solid, m.p. = 56.9 ± 4.6 °C. ^1^H-NMR (500.16 MHz, CDCl_3_) (Figure S12, Supplementary Materials): δ (ppm) = 7.92 (1H, td, *J* = 7.6 and 1.8 Hz, HAr-6′); 7.56–7.48 (1H, m, HAr-4′); 7.21 (1H, td, *J* = 7.6 and 1.2 Hz, HAr-3′); 7.13 (1H, ddd, *J* = 10.7, 7.6 and 0.9 Hz, HAr-5′); 5.40–5.39 (1H, m, H-3); 3.87–3.76 (1H, m, H-23a); 3.75–3.39 (2H, m, H-22, H-23b); 2.71 (1H, dd, *J* = 12.5 and 3.1 Hz, H-5); 2.31 (1H, dd, *J* = 13.1 and 4.6 Hz, H-7α); 0.917 (3H, d, *J* = 6.4 Hz, H-21); 0.776 (3H, s, H-19); 0.676 (3H, s, H-18). ^13^C-NMR (125.77 MHz, CDCl_3_) (Figure S12, Supplementary Materials): δ (ppm) = 212.12 (C-6); 163.89 (d, ^3^*J*_CF_ = 3,6 Hz, CO_2_-Ar); 161.93 (d, ^1^*J*_CF_ = 259.1 Hz, C2′-Ar); 134.49 (d, ^3^*J*_CF_ = 8.4 Hz, C4′-Ar); 132.31 (d, ^3^*J*_CF_ = 0.9 Hz, C6′-Ar); 124.13 (d, ^4^*J*_CF_ = 3.6 Hz, C5′-Ar); 117.17 (d, ^2^*J*_CF_ = 9.6 Hz, C1′-Ar); 116.99 (d, ^2^*J*_CF_ = 21.6 Hz, C3′-Ar); 75.56 (C-22); 70.33 (C-3); 61.15 (C-23); 56.67 (C-14); 53.85 (C-17); 52.79 (C-9); 52.45 (C-5); 46.78 (C-7); 43.47 (C-13); 42.93 (C-10); 41.41 (C-20); 39.51 (C-12); 38.09 (C-8); 32.55 (C-1); 27.72 (C-16); 25.49 (C-2); 25.20 (C-4); 23.94 (C-15); 21.21 (C-11); 12.73 (C-21); 12.61 (C-19); 12.00 (C-18). ^19^F-NMR (470.62 MHz, CDCl_3_) δ (ppm) = −108,91 (s, 1F). HRMS (API+) (Figure S19, Supplementary Materials): calculated for C_30_H_42_O_5_F ([M + H]^+^) 501.3016, found 501.3015.

Compound **19b** was obtained as a colorless solid, m.p. = 75.4 ± 1.5 °C. ^1^H-NMR (500.16 MHz, CDCl_3_) (Figure S13, Supplementary Materials): δ (ppm) = 7.92 (1H, td, *J* = 7.6 and 1.8 Hz, HAr-6′); 7.54–7.49 (1H, m, HAr-4′); 7.21 (1H, td, *J* = 7.6 and 1.2 Hz, HAr-3′); 7.13 (1H, ddd, *J* = 10.7, 7.6, and 0.9 Hz, HAr-5′); 5.40–5.39 (1H, m, H-3); 3.84–3.76 (1H, m, H-23a); 3.71–3.69 (1H, m, H-23b); 3.51 (1H, t, *J* = 10.2 Hz, H-22); 2.70 (1H, dd, *J* = 12.5 and 3.1 Hz, H-5); 2.31 (1H, dd, *J* = 13.1 and 4.6 Hz, H-7α); 0.954 (3H, d, *J* = 6.7 Hz, H-21); 0.777 (3H, s, H-19); 0.681 (3H, s, H-18). ^13^C-NMR (125.77 MHz, CDCl_3_) (Figure S13, Supplementary Materials): δ (ppm) = 211.97 (C-6); 163.89 (d, ^3^*J*_CF_ = 3.6 Hz, CO_2_-Ar); 160.87 (d, ^1^*J*_CF_ = 259.1 Hz, C2′-Ar); 134.48 (d, ^3^*J*_CF_ = 8.4 Hz, C4′-Ar); 132.34 (d, ^3^*J*_CF_ = 0.9 Hz, C6′-Ar); 124.13 (d, ^4^*J*_CF_ = 3.6 Hz, C5′-Ar); 118.37 (d, ^2^*J*_CF_ = 9.6 Hz, C1′-Ar); 117.04 (d, ^2^*J*_CF_ = 21.6 Hz, C3′-Ar); 73.90 (C-22); 70.32 (C-3); 62.52 (C-23); 56.37 (C-14); 53.95 (C-17); 52.90 (C-9); 52.79 (C-5); 46.78 (C-7); 43.48 (C-13); 41.38 (C-10); 40.15 (C-20); 39.46 (C-12); 38.04 (C-8); 32.57 (C-1); 27.47 (C-16); 25.48 (C-2); 25.20 (C-4); 24.11 (C-15); 21.19 (C-11); 13.13 (C-21); 12.62 (C-19); 11.85 (C-18). ^19^F-NMR (470.62 MHz, CDCl_3_) δ (ppm) = -108,90 (s, 1F). HRMS (API+) (Figure S20, Supplementary Materials): calculated for C_30_H_42_O_5_F ([M + H]^+^) 501.3016, found 501.3014.

### 3.2. Biological

#### Rice Lamina Inclination Test (RLIT)

The biological activity of the growth of the compounds was evaluated by the rice lamina inclination test [55,56], according to a previously described procedure [38], and using the same a Zafiro cultivar (*Oryza sativa*) provided by the Institute of Agricultural Research (INIA-Quilamapu-Chile) as previous studies.

The seeds were sown and cultivated until the seedlings presenting the second internode of the rice blade were selected for cutting. Six segments per treatment were incubated in Petri dishes containing 60 mL of distilled water, and the amount of test compound (BRs analogs **18a, 18b, 19a, 19b**, **20a,** and **20b** and positive control (**1**)) needed to reach final concentrations equal to 1 × 10^−8^ M; 1 × 10^−7^ M; and 1 × 10^−6^ M. The negative control only contained sterile distilled water. All treatments were incubated by 48 h at 25 °C in darkness, and the angles developed between the blade and the sheath were measured. Each experiment was performed by duplicate.

Results were expressed as mean ± standard deviation (SD) using twelve angle measurements. Statistical analysis was done using a statistical package Excel by applying mean values using one-way ANOVA with the post-hoc least square differences (LSD) test to determine if there was a significant difference between the positive control and the treatments. A *P* value of less than 0.05 was considered significant.

## 4. Conclusions

Brassinosteroid 24-norcholane type analogs conjugated at C-3 and configurations *S* and *R* on the C-22 carbon of the side chain have been synthesized and characterized. The synthesis uses hyodeoxycholic acid as the starting material, and epimers with different configuration at C-22 are obtained. These epimers have been separated, and their growth-promoting activity was measured using RLIT. The results show that the esterification of BRs analog at C-3 has no effect on the biological activity of synthetic analogs. This suggest that reducing activity by esterification at C-3 requires a long chain carboxylic acid. In addition, the presence of a hydroxyl group at C-3 is not an essential structural feature for activity. This result confirms previous SAR where it has been proposed that activity is not determined by the presence or absence of specific groups in the BR structure.

## Figures and Tables

**Table 1:** Differences in ^1^H- and ^13^C-NMR chemical shifts for H-3, H-6, C-3, and C-6 observed for compounds **24**–**27**.

Compound	H-3 (δ_H_ ppm)	H-6 (δ_H_ ppm)	C-3 (δ_C_ ppm)	C-6 (δ_C_ ppm)
24	3.48–3.42	4.02–3.96	71.72	67.63
25	-	4.15–4.10	212.63	67.73
26	-	-	208.65	210.82
27	3.70–3.62	-	70.18	213.89

**Table 2:** Comparison between signals of ^1^H- (500.1 MHz, CDCl_3_) and ^13^C- (125.8 MHz, CDCl_3_) NMR for H/C21, H/C22, and H/C23a-b, for the epimers **18a** and **18b**.

H/C Signal	Compound 18a	Compound 18b
H-21	0.921 ppm (3H, d, *J* = 6.7 Hz)	0.953 ppm (3H, d, *J* = 7.0 Hz)
H-22	3.66–3.61 ppm (1H, m)	3.51 ppm (1H, t, *J* = 10.2 Hz)
H-23a	3.80 ppm (1H, ddd, *J* = 8.9, 3.3 and 1.2 Hz)	3.83–3.76 ppm (1H, m)
H-23b	3.52 ppm (1H, dd, *J* = 10.8 and 3.3 Hz)	3.69–3.57 ppm (1H, m)
C21	12.73 ppm	13.15 ppm
C22	74.14 ppm	73.92 ppm
C23	66.16 ppm	62.54 ppm

**Table 3:** Comparison between signals of ^1^H- (500.1 MHz, CDCl_3_) and ^13^C- (125.8 MHz, CDCl_3_) NMR for H/C21, H/C22, and H/C23a-b, for the epimers **19a** and **19b**.

H/C Signal	Compound 19a	Compound 19b
H-21	0.917 ppm (3H, d, *J* = 6.4 Hz)	0.954 ppm (3H, d, *J* = 6.7 Hz)
H-22	3.75–3.39 ppm (1H, m)	3.51 ppm (1H, t, *J* = 10.2 Hz)
H-23a	3.87–3.76 ppm (1H, m)	3.84–3.76 ppm (1H, m)
H-23b	3.75–3.39 ppm (1H, m)	3.71–3.59 ppm (1H, m)
C21	12.73 ppm	13.13 ppm
C22	75.56 ppm	73.90 ppm
C23	61.15 ppm	62.52 ppm

**Table 4:** Comparison between BRs C-3 conjugated 24-norcholane and free 24-norcholane type analogs on lamina inclination of rice seedlings.

Bending Angle between Laminae and Sheaths (Degrees ± SD)			
Compounds	1 × 10^−8^ M	1 × 10^−7^ M	1 × 10^−6^ M
**1** (C+)	31 ± 11	41 ± 4.5	70 ± 7.6
**18a**	61 ± 6.3 *	68 ± 9.6 *	46 ± 7.5 *
**18b**	64 ± 3.3 *	48 ± 2.9	14 ± 4.8 *
**19a**	43 ± 5.0 *	58 ± 2.4 *	68 ± 9.6
**19b**	68 ± 5.0 *	61 ± 2.5 *	30 ± 0.0 *
**20a** ^†^	45 ± 9.5 *	31 ± 5.0	24 ± 5.8 *
**20b** ^†^	35 ± 3.0	60 ± 3.0 *	62 ± 12
Control (C−)	7 ± 5.0		

^†^ Data previously obtained and reported in reference [38]. Brassinolide (**1**) was used as positive control. The negative control only contained sterile distilled water. These values represent the mean ± standard deviation of two independent experiments with at least six replicates each (*n* = 12). (*) Represents experiments with a significant difference between positive control (**1**) and analog treatments at *p* < 0.05 significance level (least square differences (LSD) *t*-test).

## Data Availability

The data presented in this study are available in Supplementary Material.

## Supplementary Materials

The following are available online, Figure S1: NMR spectra of 3α,6α-diacetoxy-5β-cholan-24-oic acid **(22**), Figure S2: NMR spectra of 24-nor-5β-cholan-22-ene-3α,6α-diyl diacetate (**23**), Figure S3: NMR spectra of 24-nor-5β-chol-22-ene-3α,6α-diol (**24**), Figure S4: NMR spectra of 6α-hydroxy-24-nor-5β-chol-22-en-3-one (**25**), Figure S5: NMR spectra of 24-nor-5β-chol-22-ene-3,6-dione (**26**), Figure S6: NMR spectra of 3α-hydroxy-24-nor-5β-chol-22-en-6-one (**27**), Figure S7: NMR spectra of 3α-hydroxy-24-nor-5β-chol-22-en-6-one (**28**), Figure S8: NMR spectra of 6-oxo-24-nor-5β-chol-22-en-3α-yl 4-methylbenzoate (**29**), Figure S9: NMR spectra of 6-oxo-24-nor-5β-chol-22-en-3α-yl 2-fluorobenzoate (**30**), Figure S10: NMR spectra of (22*R*)-22,23-dihydroxy-6-oxo-24-nor-5β-cholan-3α-yl 4-methylbenzoate (**18a**), Figure S11: NMR spectra of (22*S*)-22,23-dihydroxy-6-oxo-24-nor-5β-cholan-3α-yl 4-methylbenzoate (**18b**), Figure S12: NMR spectra of (22*R*)-22,23-dihydroxy-6-oxo-24-nor-5β-cholan-3α-yl 2-fluorobenzoate (**19a**), Figure S13: NMR spectra of (22*S*)-22,23-dihydroxy-6-oxo-24-nor-5β-cholan-3α-yl 2-fluorobenzoate (**19b**), Figure S14: HRMS (API^+^) spectrum of 3α-hydroxy-24-nor-5β-chol-22-en-6-one (**28**), Figure S15: HRMS (API^+^) spectrum of 6-oxo-24-nor-5β-chol-22-en-3α-yl 4-methylbenzoate (**29**), Figure S16: HRMS (API^+^) spectrum of 6-oxo-24-nor-5β-chol-22-en-3α-yl 2-fluorobenzoate (**30**), Figure S17: HRMS (API^+^) spectrum of (22*R*)-22,23-dihydroxy-6-oxo-24-nor-5β-cholan-3α-yl 4-methylbenzoate (**18a**), Figure S18: HRMS (API^+^) spectrum of (22*S*)-22,23-dihydroxy-6-oxo-24-nor-5β-cholan-3α-yl 4-methylbenzoate (**18b**), Figure S19: HRMS (API^+^) spectrum of (22*R*)-22,23-dihydroxy-6-oxo-24-nor-5β-cholan-3α-yl 2-fluorobenzoate (**19a**), Figure S20: HRMS (API^+^) spectrum of (22*S*)-22,23-dihydroxy-6-oxo-24-nor-5β-cholan-3α-yl 2-fluorobenzoate (**19b**).
