# Supplementary material for: Synthesis of New Brassinosteroid 24-Norcholane Type Analogs Conjugated in C-3 with Benzoate Groups
Source: Molecules. 2021 Feb 22;26(4):1173. doi: 10.3390/molecules26041173 (PMC7927124; doi:10.3390/molecules26041173)
Supplement: Supplementary file 1 [file molecules-26-01173-s001.pdf]

molecules

Article

Synthesis of New Brassinosteroid 24-Norcholane Type Analogs Conjugated in C-3 with Benzoate Groups

Karoll Ferrer [1,2], Katy Díaz [1], Miroslav Kvasnica [2,*], Andrés F. Olea [3], Mauricio Cuellar [4] and Luis Espinoza [1,*]

Departamento de Química, Universidad Técnica Federico Santa María, Avenida España 1680, 2340000 Valparaíso, Chile; karoll.ferrer.14@sansano.usm.cl (K.F.); katy.diaz@usm.cl (K.D.)

[2] Laboratory of Growth Regulators, Institute of Experimental Botany, The Czech Academy of Sciences & Department of Experimental Biology, Faculty of Science, Palacký University, Šlechtitelů 27, 78371 Olomouc, Czech Republic

[3] Instituto de Ciencias Químicas Aplicadas, Facultad de Ingeniería, Universidad Autónoma de Chile, El Llano Subercaseaux 2801, 8900000 Santiago, Chile; andres.olea@uautonoma.cl

[4] Facultad de Farmacia, Escuela de Química y Farmacia, Universidad de Valparaíso, Av. Gran Bretaña 1093, 2340000 Valparaíso, Chile, mauricio.cuellar@uv.cl

* Correspondence: kvasnica@ueb.cas.cz (M.K.); luis.espinozac@usm.cl (L.E.); Tel.: +42-07-31664627 (M.K.); +56-32-2654425 (L.E.)

Supplementary Materials: The following are available online at www.mdpi.com/xxx/s1, **Figure S1**: NMR spectra of 3α,6α-diacetoxy-5β-cholan-24-oic acid **(22)**, **Figure S2**: NMR spectra of 24-nor-5β-cholan-22-ene-3α,6α-diyl diacetate (**23**), **Figure S3**: NMR spectra of 24-nor-5β-chol-22-ene-3α,6α-diol (**24**), **Figure S4**: NMR spectra of 6α-hydroxy-24-nor-5β-chol-22-en-3-one (**25**), **Figure S5**: NMR spectra of 24-nor-5β-chol-22-ene-3,6-dione (**26**), **Figure S6**: NMR spectra of 3α-hydroxy-24-nor-5β-chol-22-en-6-one (**27**), **Figure S7**: NMR spectra of 3α-hydroxy-24-nor-5α-chol-22-en-6-one (**28**), **Figure S8**: NMR spectra of 6-oxo-24-nor-5α-chol-22-en-3α-yl 4-methylbenzoate (**29**), **Figure S9**: NMR spectra of 6-oxo-24-nor-5α-chol-22-en-3α-yl 2-fluorobenzoate (**30**), **Figure S10**: NMR spectra of (22*R*)-22,23-dihydroxy-6-oxo-24-nor-5α-cholan-3α-yl 4-methylbenzoate (**18a**), **Figure S11**: NMR spectra of (22*S*)-22,23-dihydroxy-6-oxo-24-nor-5α-cholan-3α-yl 4-methylbenzoate (**18b**), **Figure S12**: NMR spectra of (22*R*)-22,23-dihydroxy-6-oxo-24-nor-5α-cholan-3α-yl 2-fluorobenzoate (**19a**), **Figure S13**: NMR spectra of (22*S*)-22,23-dihydroxy-6-oxo-24-nor-5α-cholan-3α-yl 2-fluorobenzoate (**19b**), **Figure S14**: HRMS (API^+) spectrum of 3α-hydroxy-24-nor-5α-chol-22-en-6-one (**28**), **Figure S15**: HRMS (API^+) spectrum of 6-oxo-24-nor-5α-chol-22-en-3α-yl 4-methylbenzoate (**29**), **Figure S16**: HRMS (API^+) spectrum of 6-oxo-24-nor-5α-chol-22-en-3α-yl 2-fluorobenzoate (**30**), **Figure S17**: HRMS (API^+) spectrum of (22*R*)-22,23-dihydroxy-6-oxo-24-nor-5α-cholan-3α-yl 4-methylbenzoate (**18a**), **Figure S18**: HRMS (API^+) spectrum of (22*S*)-22,23-dihydroxy-6-oxo-24-nor-5α-cholan-3α-yl 4-methylbenzoate (**18b**), **Figure S19**: HRMS (API^+) spectrum of (22*R*)-22,23-dihydroxy-6-oxo-24-nor-5α-cholan-3α-yl 2-fluorobenzoate (**19a**), **Figure S20**: HRMS (API^+) spectrum of (22*S*)-22,23-dihydroxy-6-oxo-24-nor-5α-cholan-3α-yl 2-fluorobenzoate (**19b**).

^{1}H-NMR

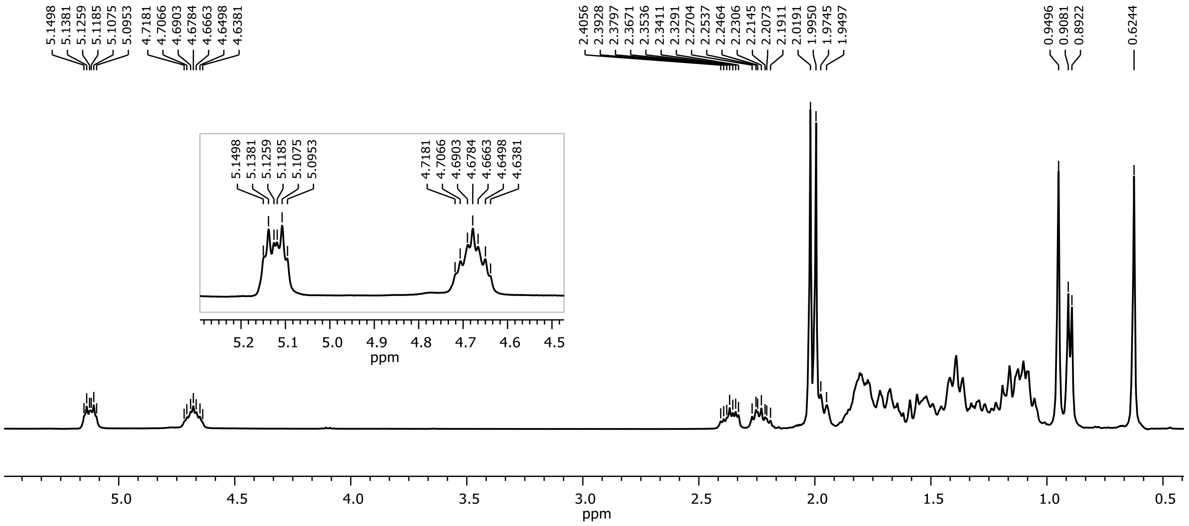

^{13}C-NMR

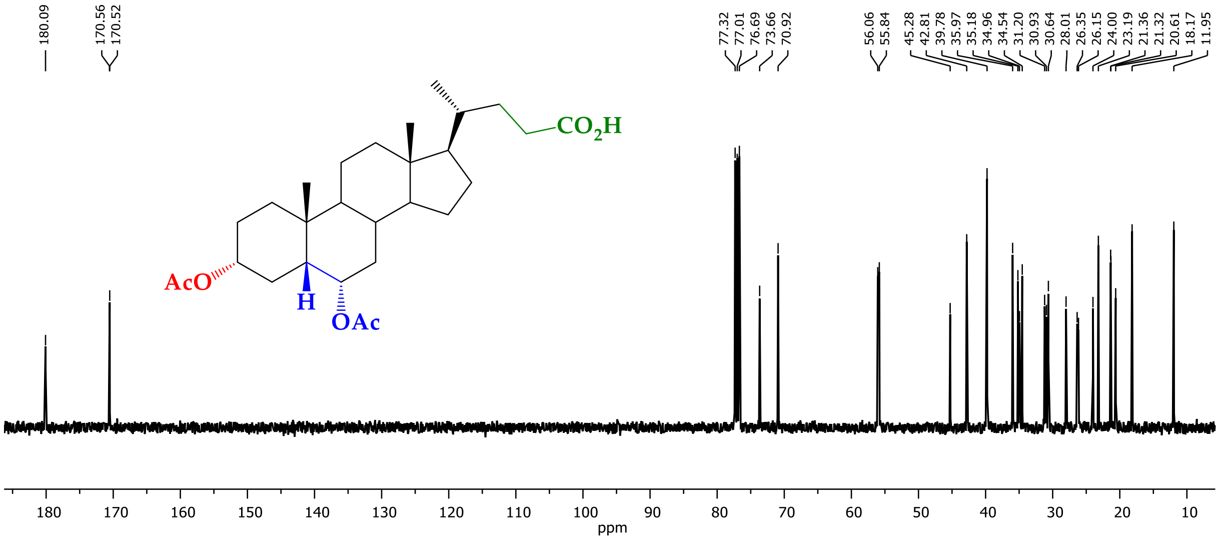

Figure S1: NMR spectra of 3α,6α-diacetoxy-5β-cholan-24-oic acid **(22)**

^{1}H-NMR

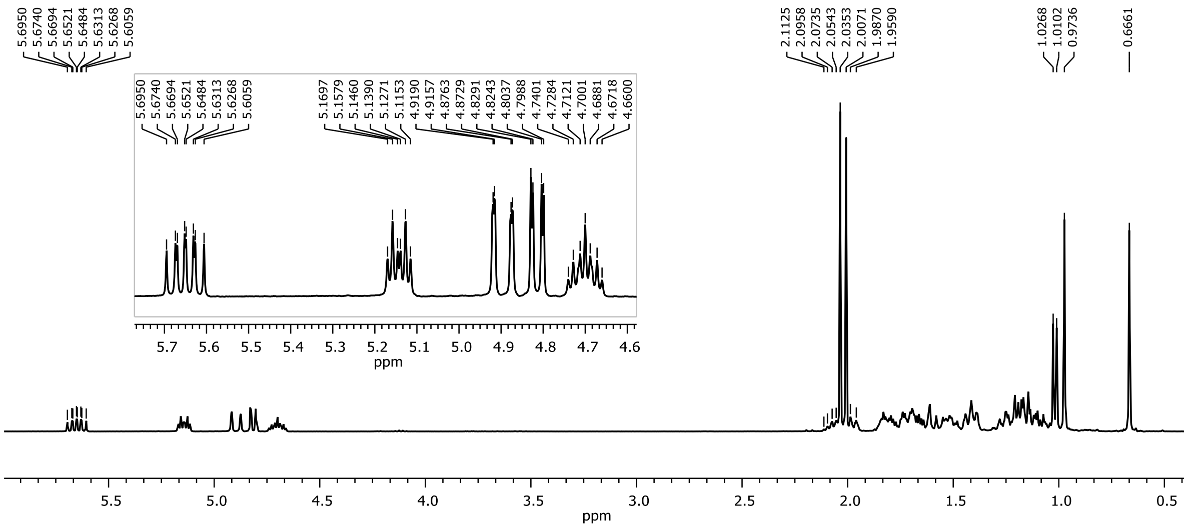

^{13}C-NMR

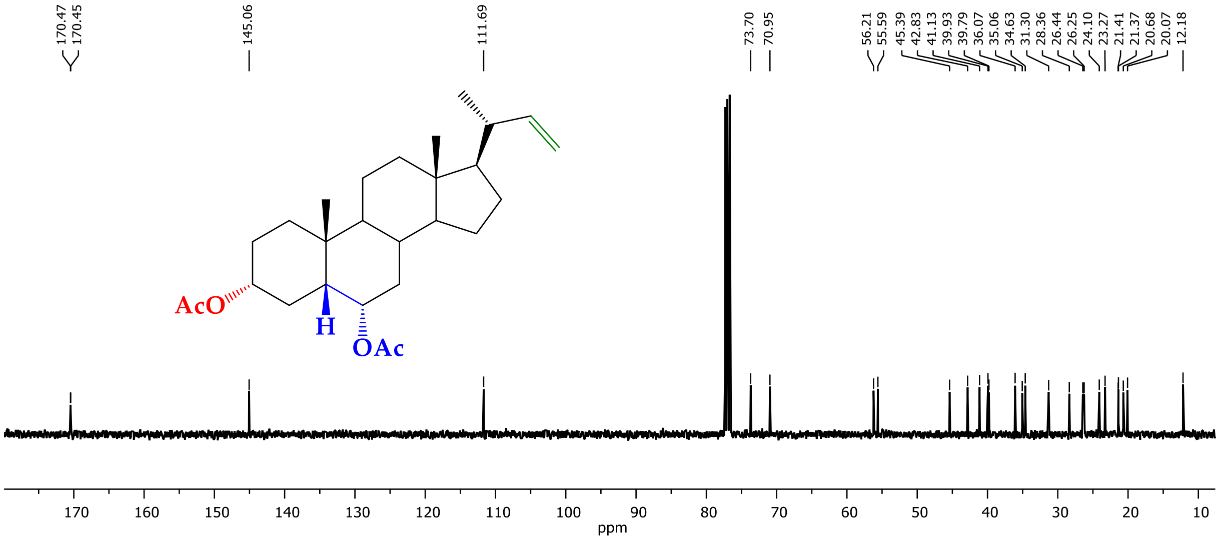

Figure S2: NMR spectra of 24-nor-5β-cholan-22-ene-3α,6α-diyl diacetate (**23**)

^{1}H-NMR

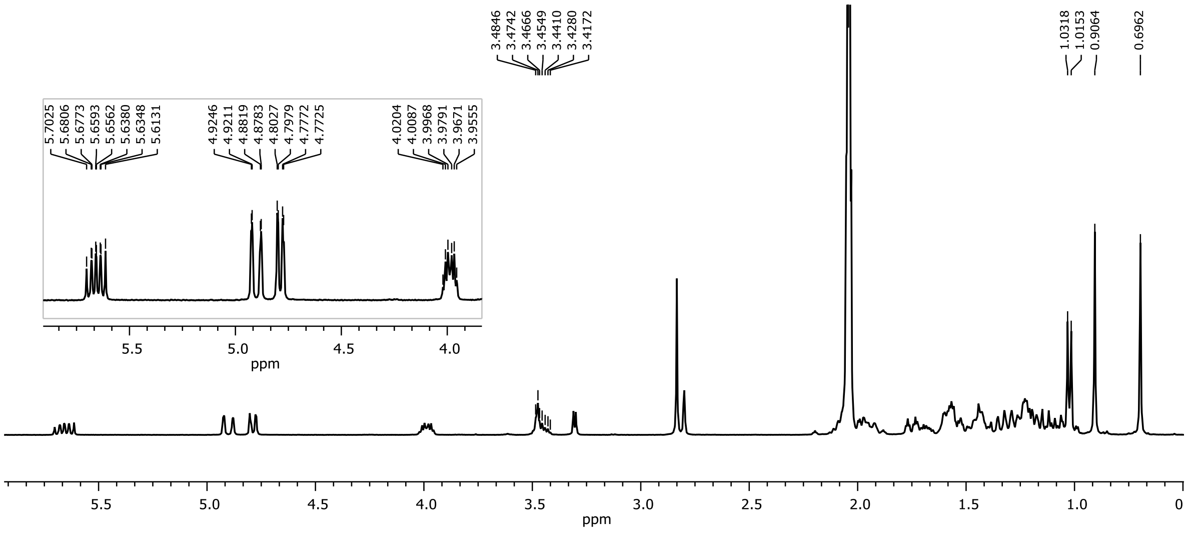

^{13}C-NMR

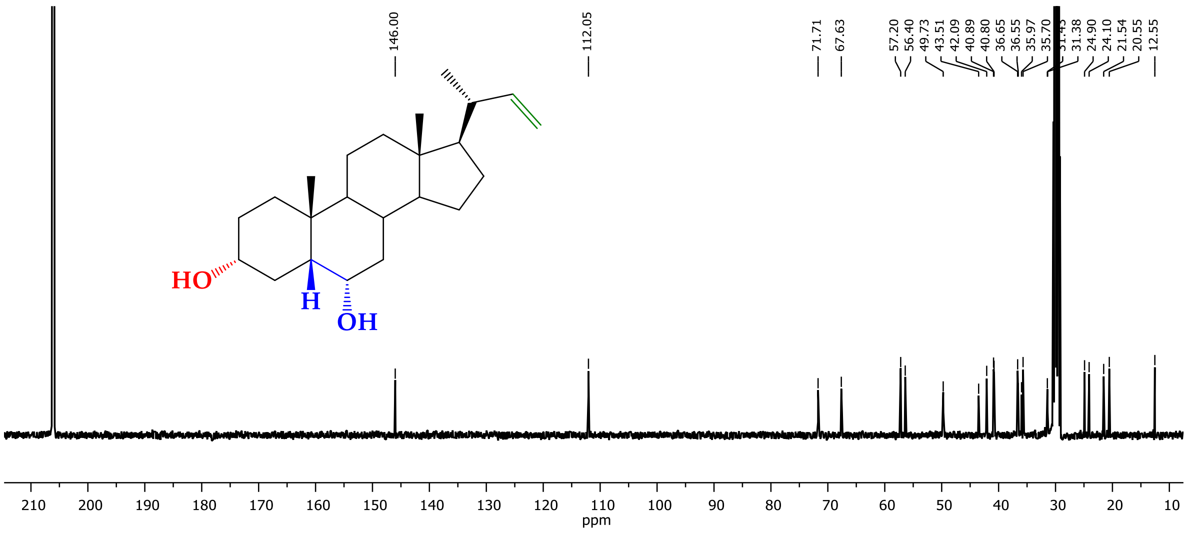

Figure S3: NMR spectra of 24-nor-5β-chol-22-ene-3α,6α-diol (**24**)

^{1}H-NMR

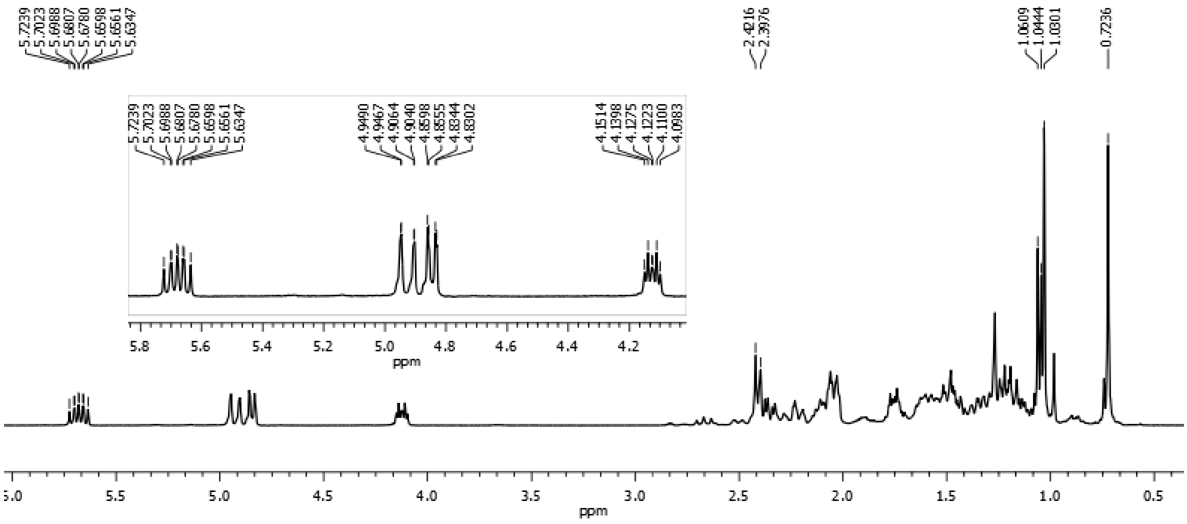

^{13}C-NMR

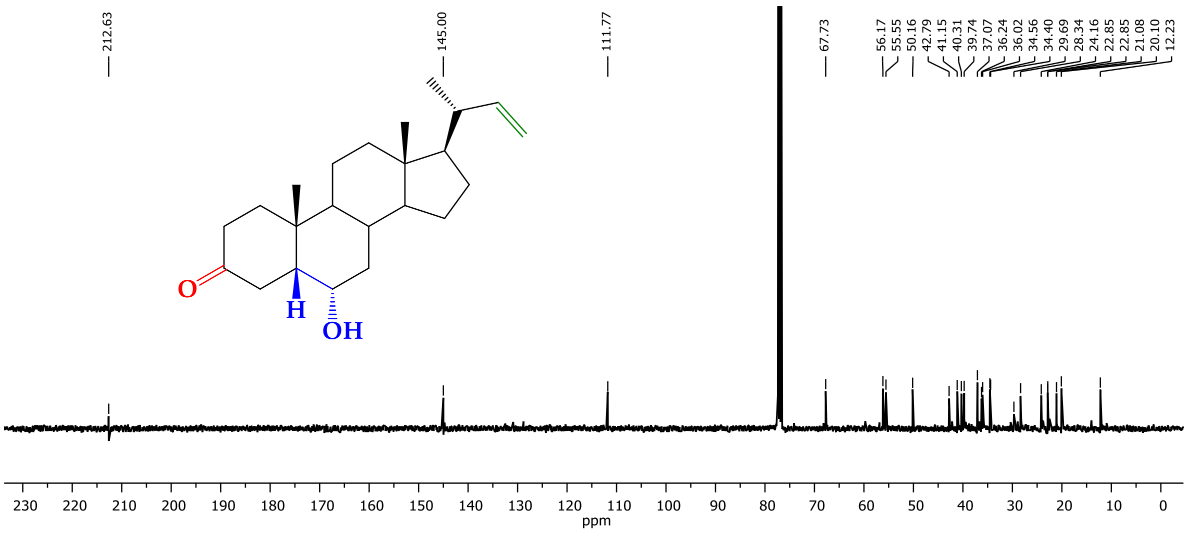

Figure S4: NMR spectra of 6α-hydroxy-24-nor-5β-chol-22-en-3-one (**25**)

^{1}H-NMR

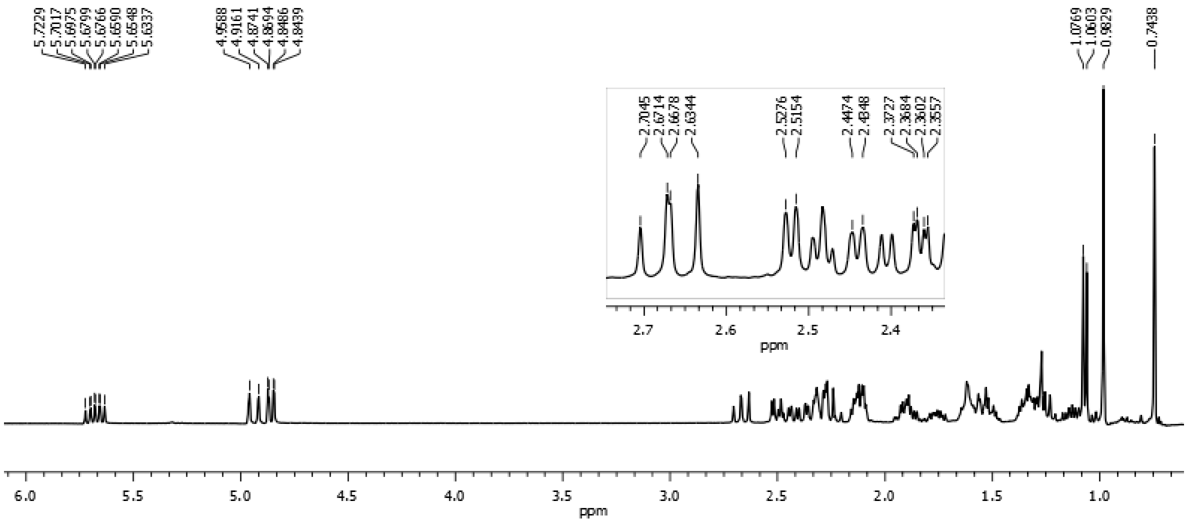

^{13}C-NMR

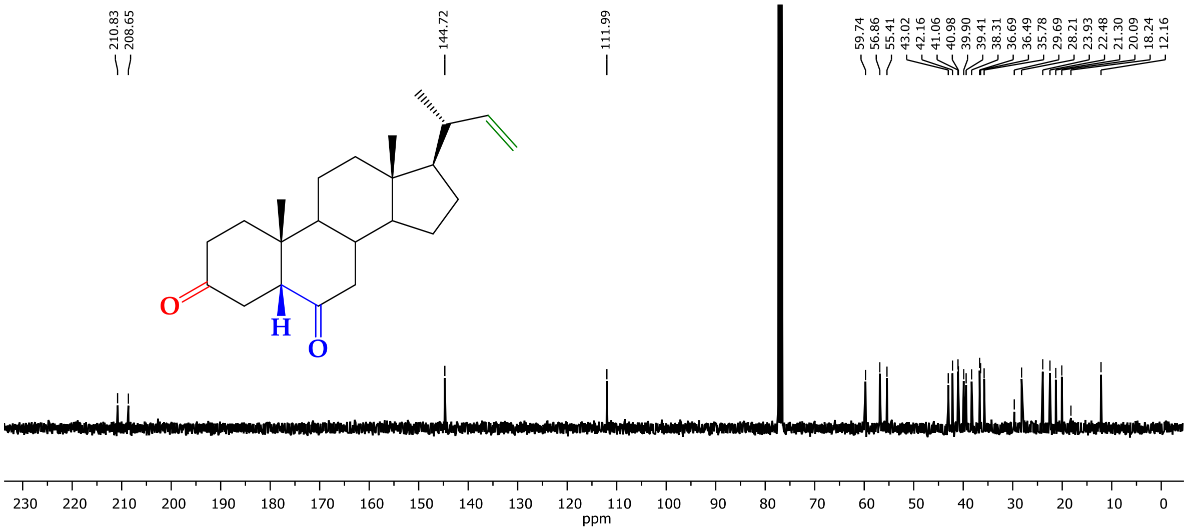

Figure S5: NMR spectra of 24-nor-5β-chol-22-ene-3,6-dione (**26**)

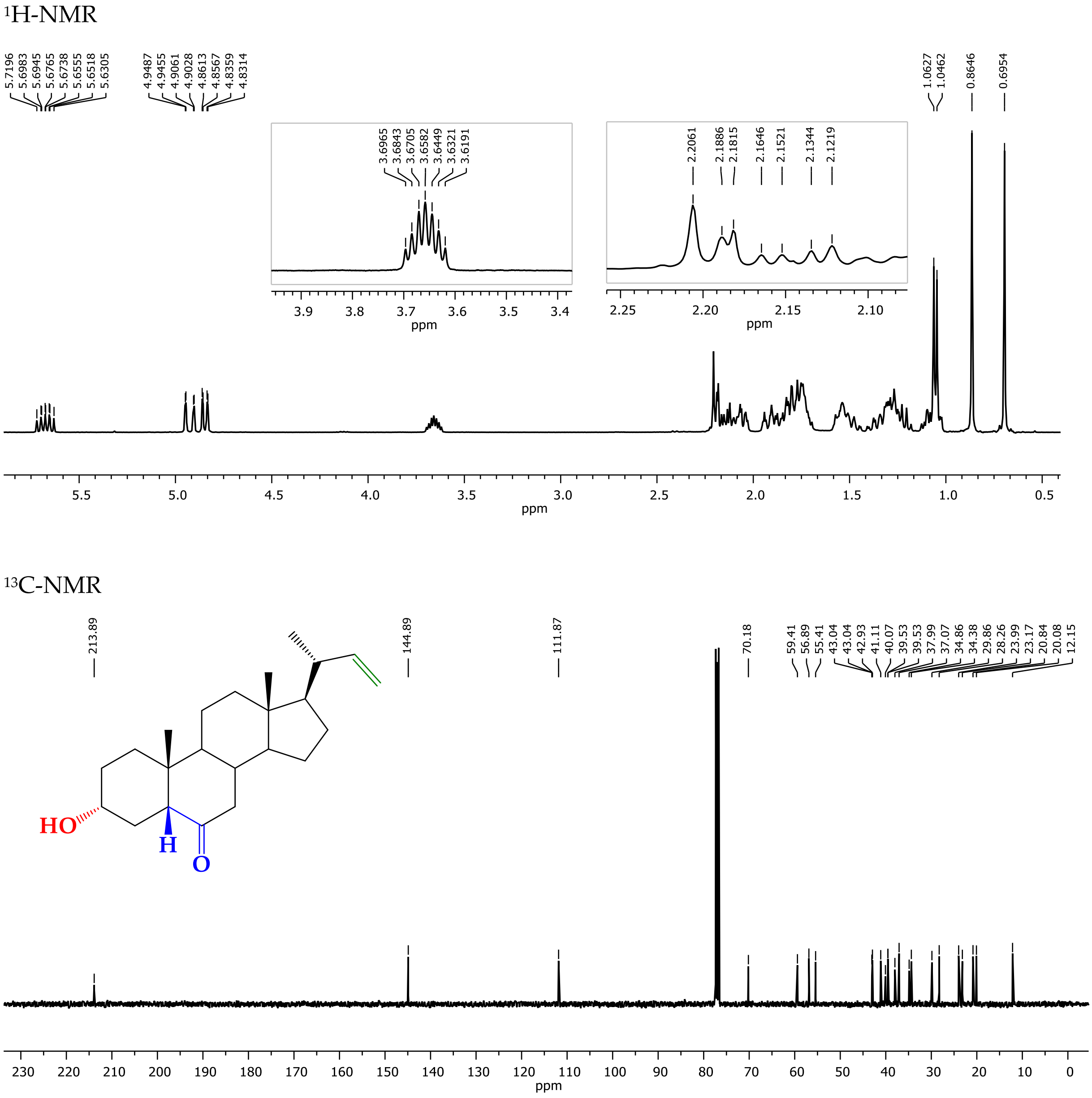

Figure S6: NMR spectra of 3α-hydroxy-24-nor-5β-chol-22-en-6-one (**27**)

^{1}H-NMR

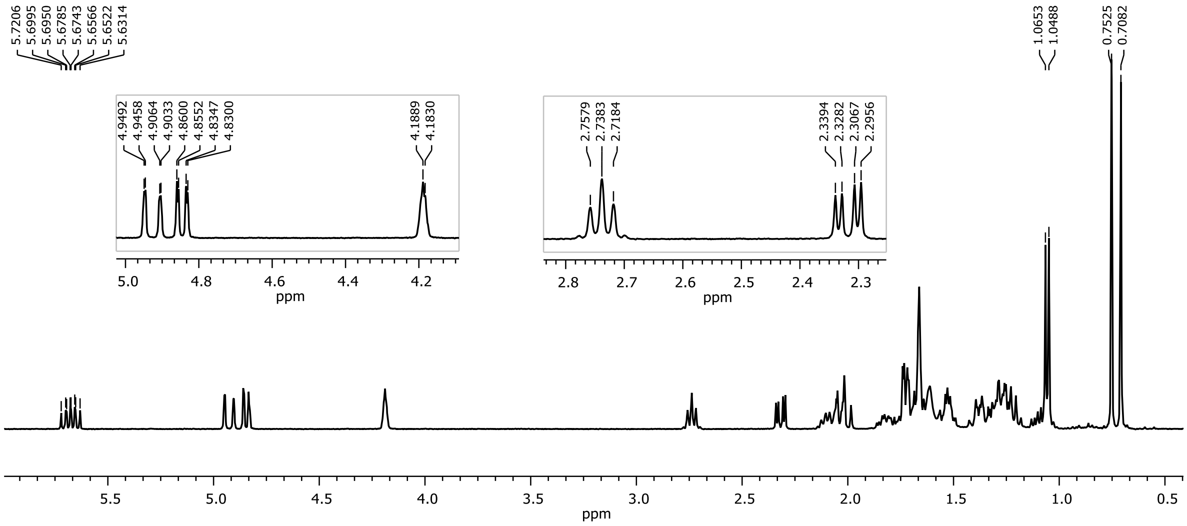

^{13}C-NMR

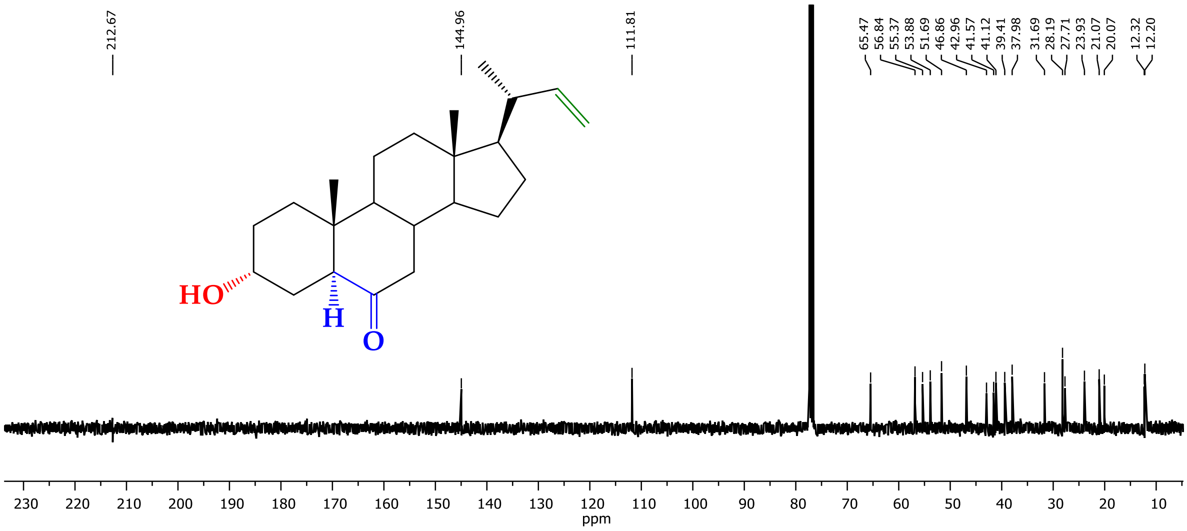

Figure S7: NMR spectra of 3α-hydroxy-24-nor-5α-chol-22-en-6-one (**28**)

^{1}H-NMR

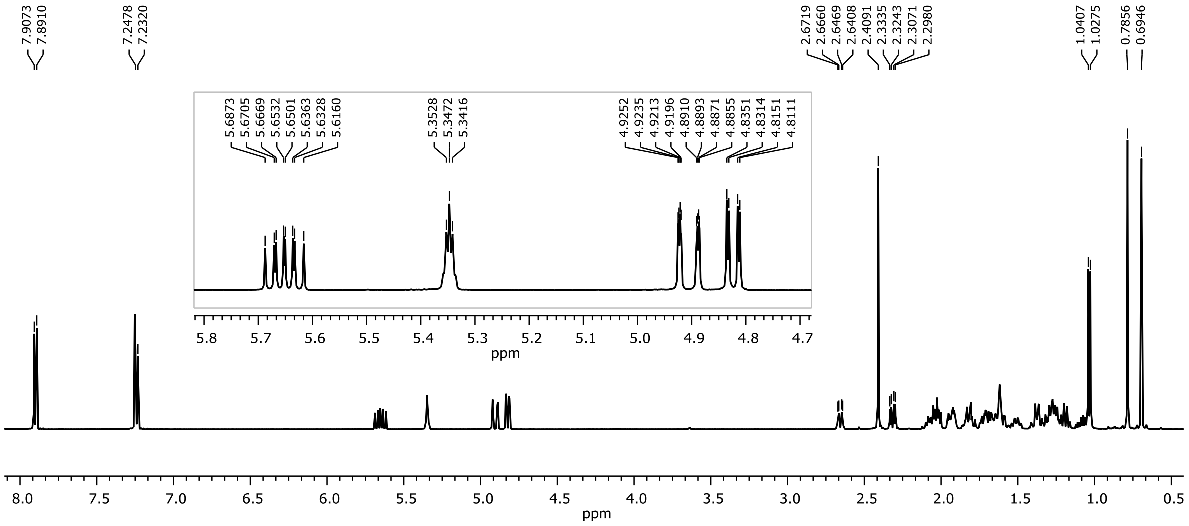

^{13}C-NMR

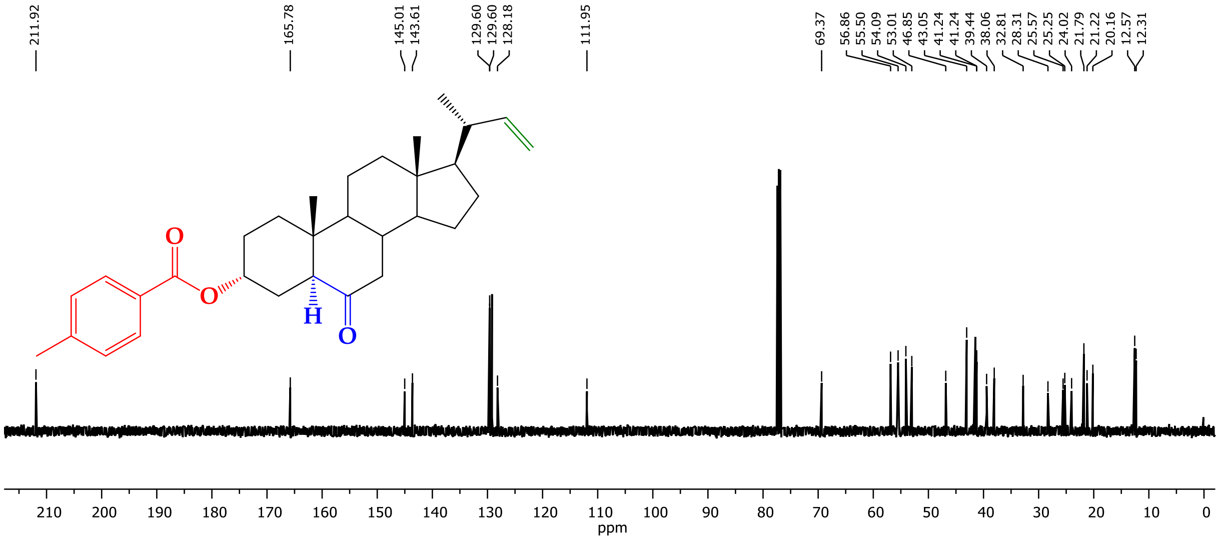

Figure S8: NMR spectra of 6-oxo-24-nor-5α-chol-22-en-3α-yl 4-methylbenzoate (**29**)

^{1}H-NMR

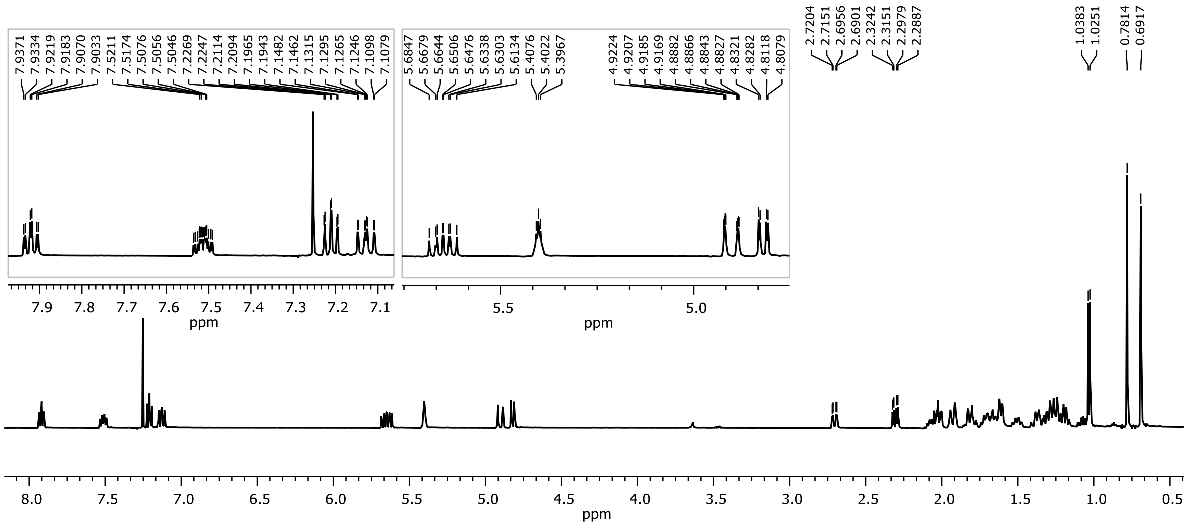

^{13}C-NMR

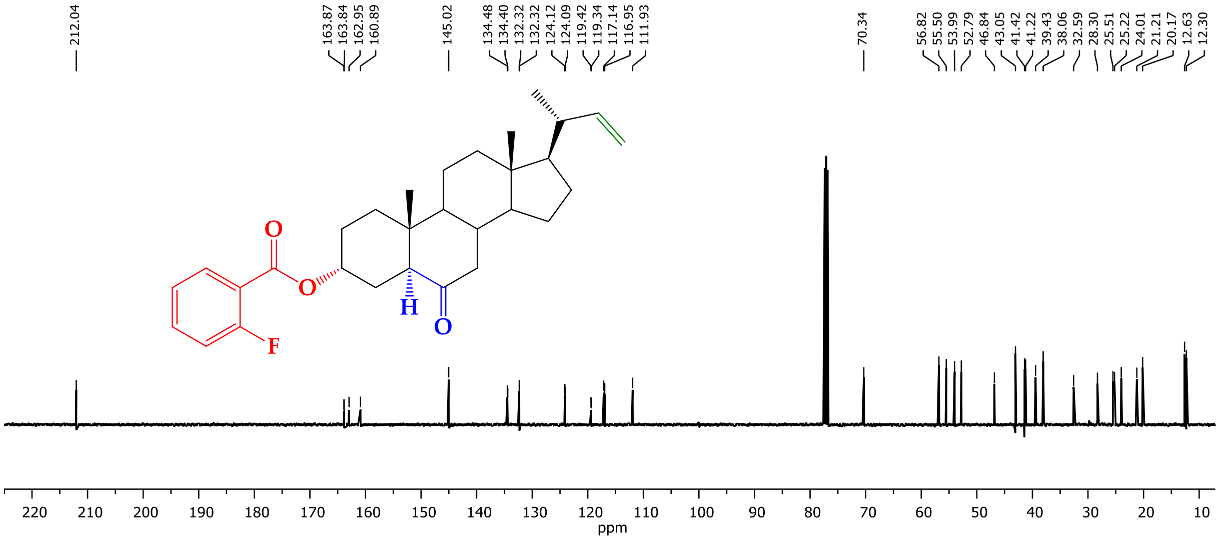

Figure S9: NMR spectra of 6-oxo-24-nor-5α-chol-22-en-3α-yl 2-fluorobenzoate (**30**)

^{1}H-NMR

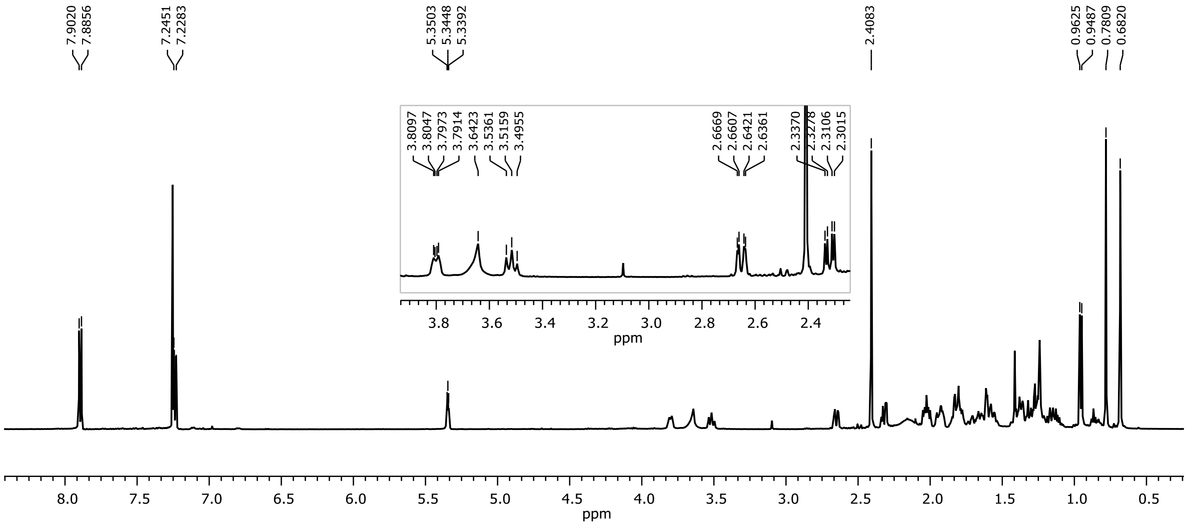

^{13}C-NMR

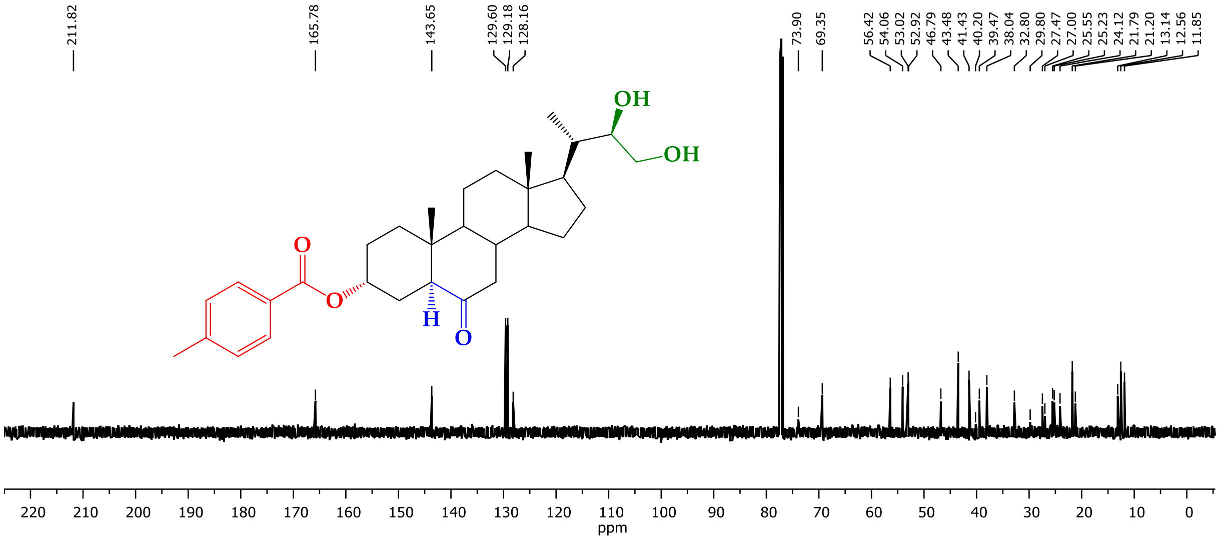

Figure S10: NMR spectra of (22*R*)-22,23-dihydroxy-6-oxo-24-nor-5α-cholan-3α-yl 4-methylbenzoate (**18a**)

^{1}H-NMR

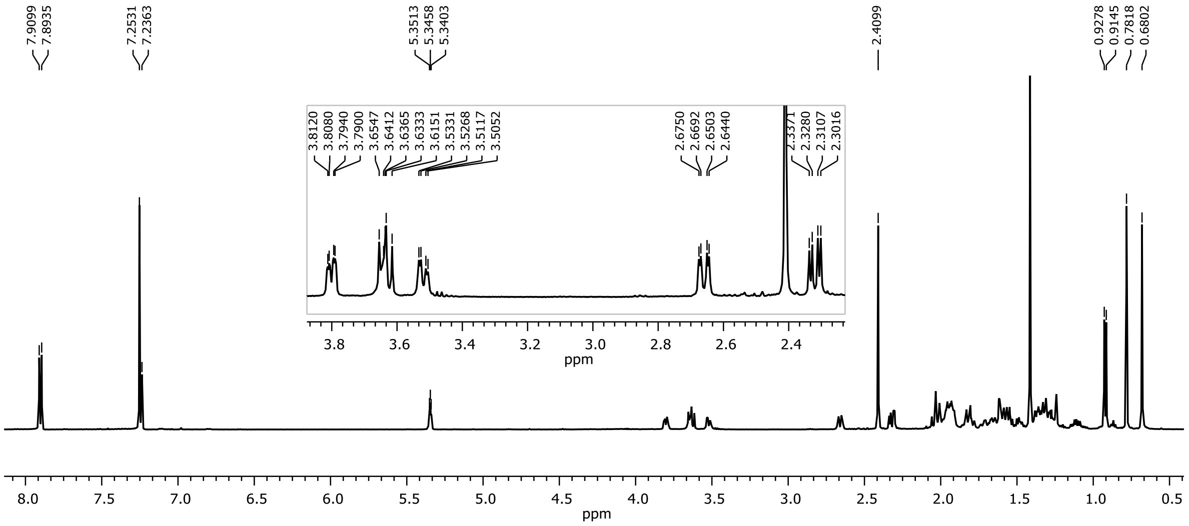

^{13}C-NMR

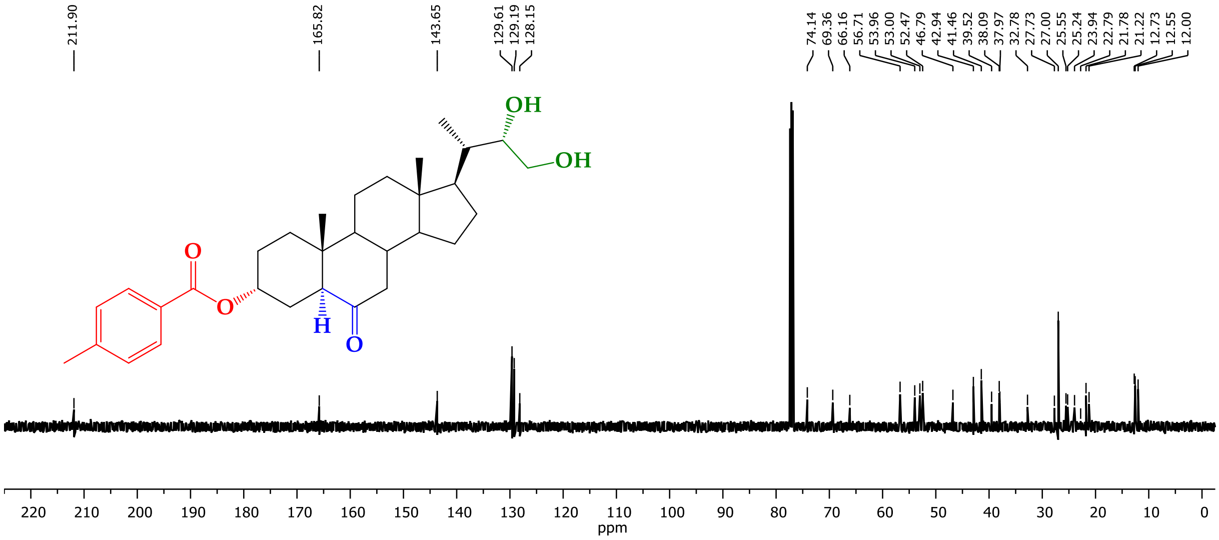

Figure S11: NMR spectra of (22*S*)-22,23-dihydroxy-6-oxo-24-nor-5α-cholan-3α-yl 4-methylbenzoate (**18b**)

^{1}H-NMR

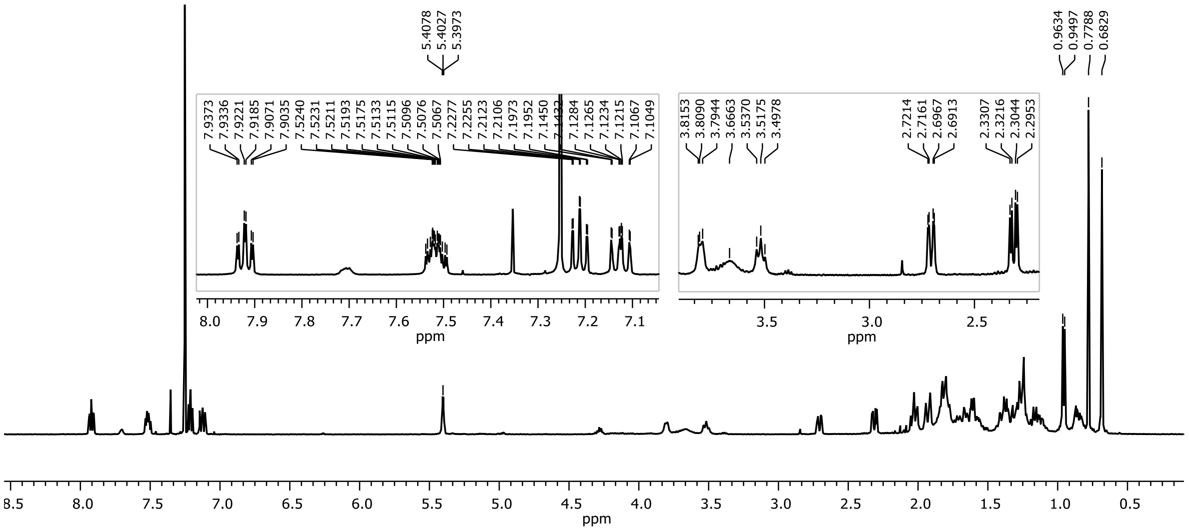

^{13}C-NMR

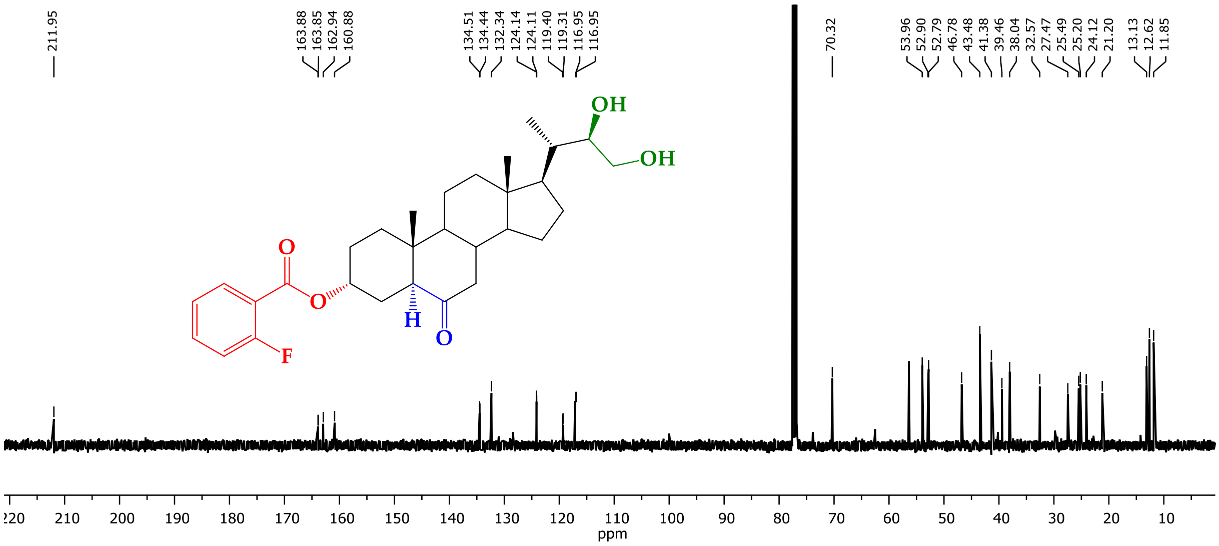

Figure S12: NMR spectra of (22*R*)-22,23-dihydroxy-6-oxo-24-nor-5α-cholan-3α-yl 2-fluorobenzoate (**19a**)

^{1}H-NMR

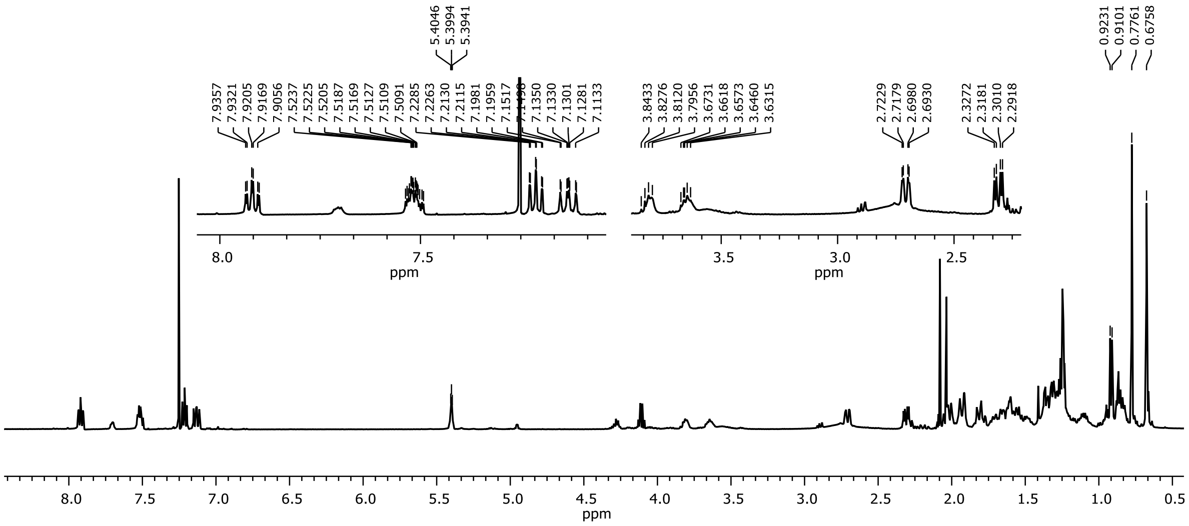

^{13}C-NMR

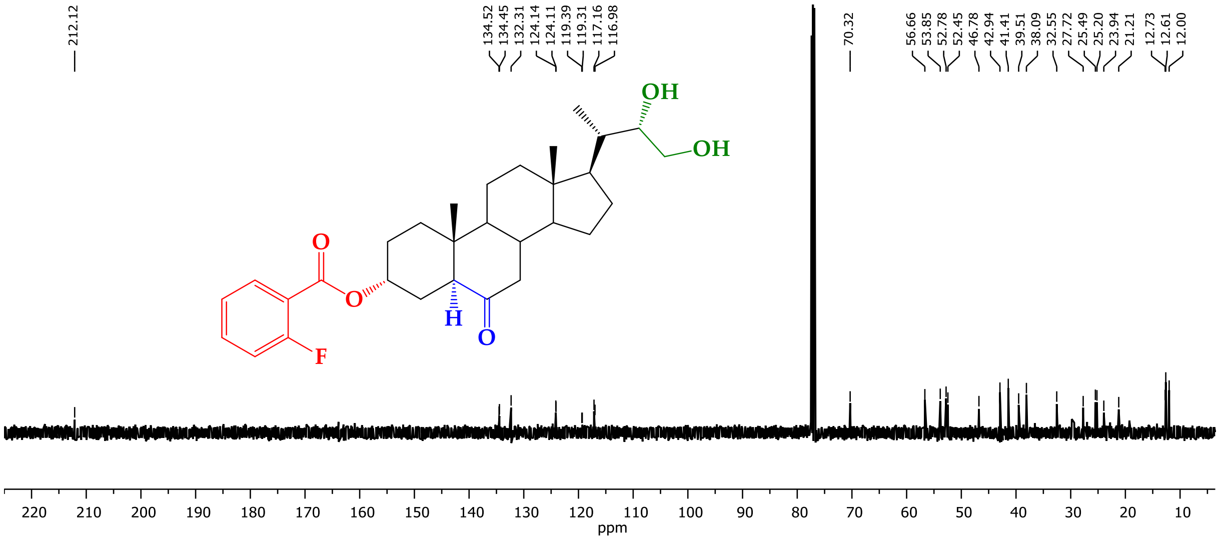

Figure S13: NMR spectra of (22*S*)-22,23-dihydroxy-6-oxo-24-nor-5α-cholan-3α-yl 2-fluorobenzoate (**19b**)

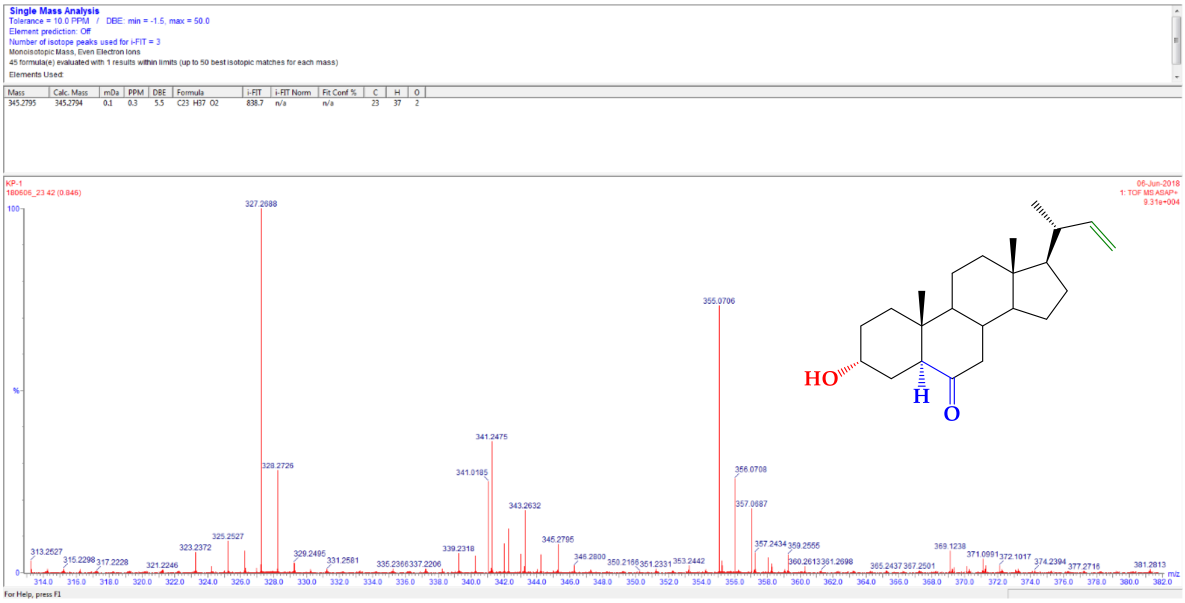

Figure S14: HRMS (API$^+$) spectrum of 3α-hydroxy-24-nor-5α-chol-22-en-6-one (**28**)

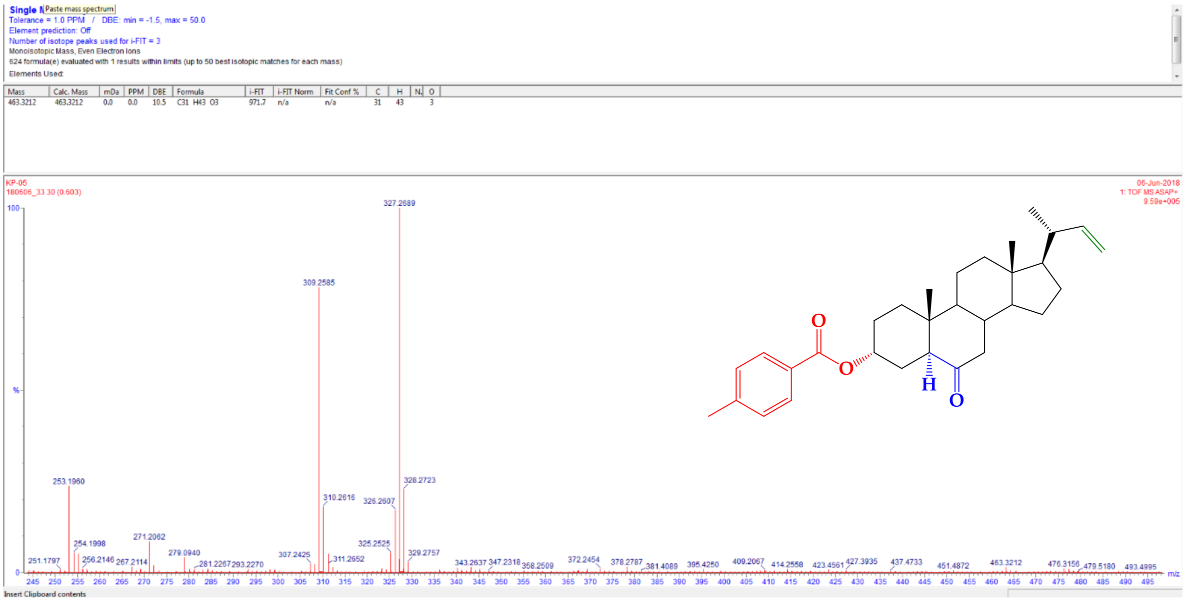

Figure S15: HRMS (API$^+$) spectrum of 6-oxo-24-nor-5α-chol-22-en-3α-yl 4-methylbenzoate (**29**)

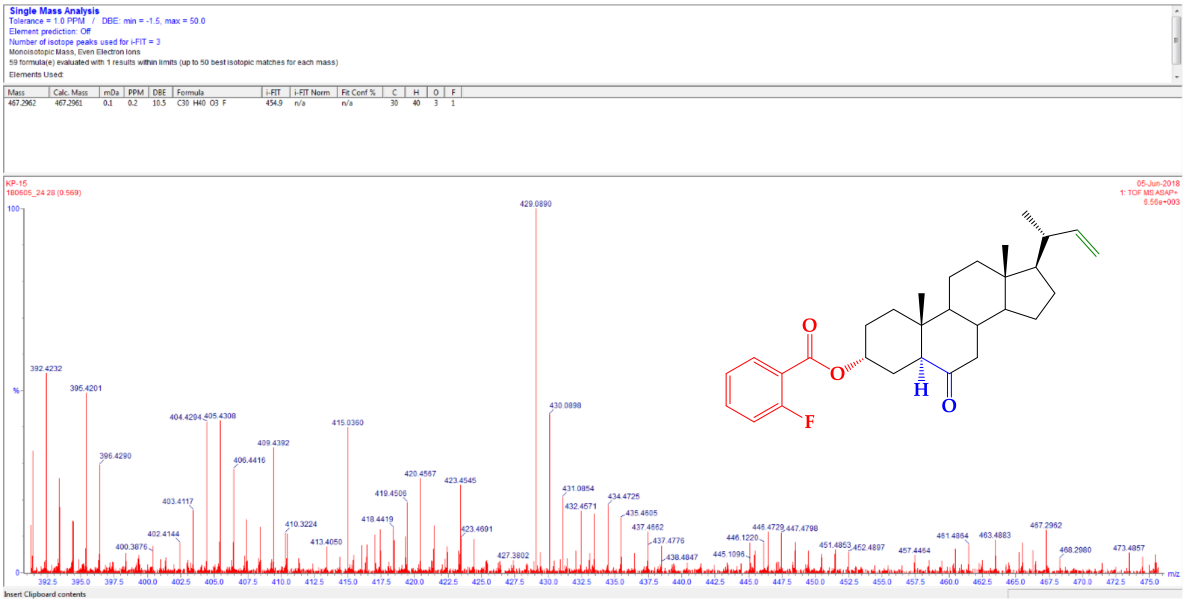

Figure S16: HRMS (API+) spectrum of 6-oxo-24-nor-5α-chol-22-en-3α-yl 2-fluorobenzoate (**30**)

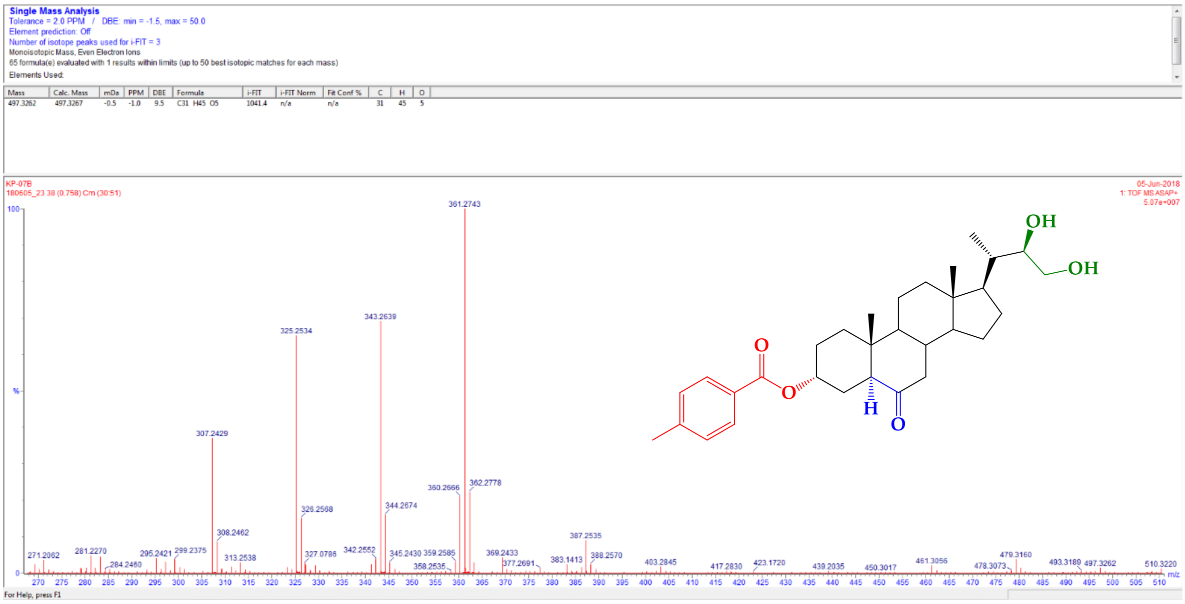

Figure S17: HRMS (API+) spectrum of (22*R*)-22,23-dihydroxy-6-oxo-24-nor-5α-cholan-3α-yl 4-methylbenzoate (**18a**)

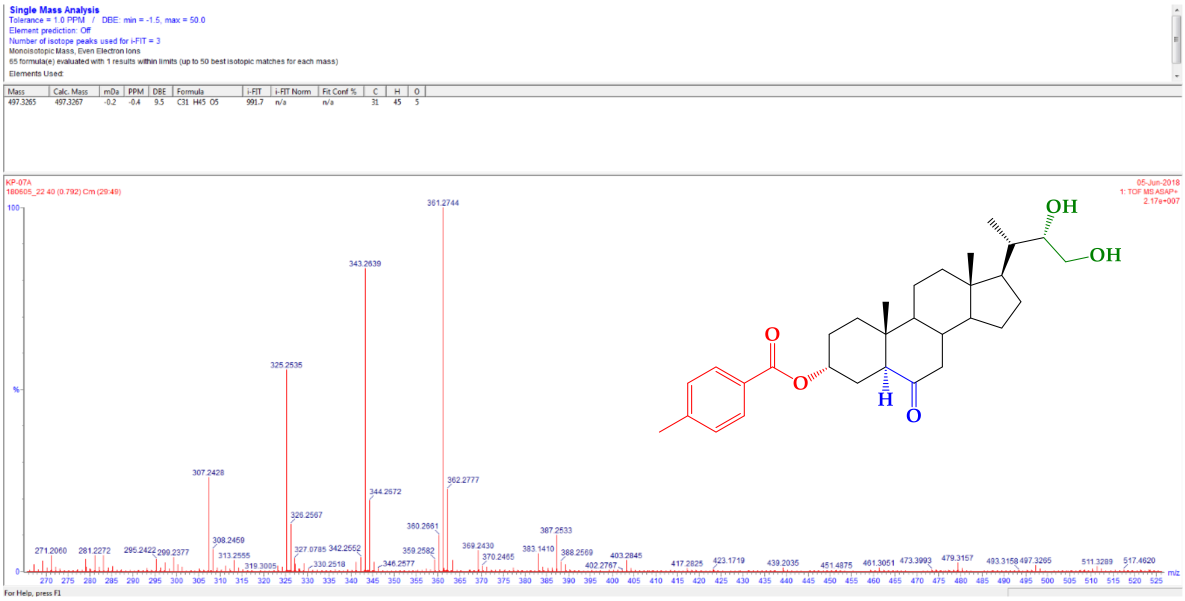

Figure S18: HRMS (API$^+$) spectrum of (22*S*)-22,23-dihydroxy-6-oxo-24-nor-5α-cholan-3α-yl 4-methylbenzoate (**18b**)

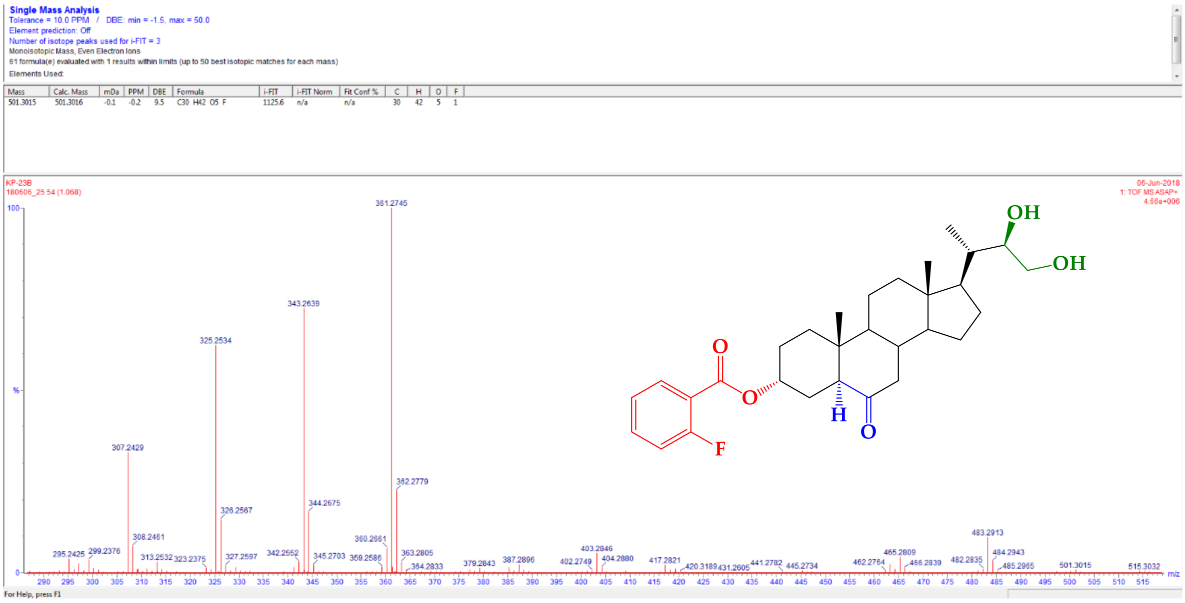

Figure S19: HRMS (API$^+$) spectrum of (22*R*)-22,23-dihydroxy-6-oxo-24-nor-5α-cholan-3α-yl 2-fluorobenzoate (**19a**)

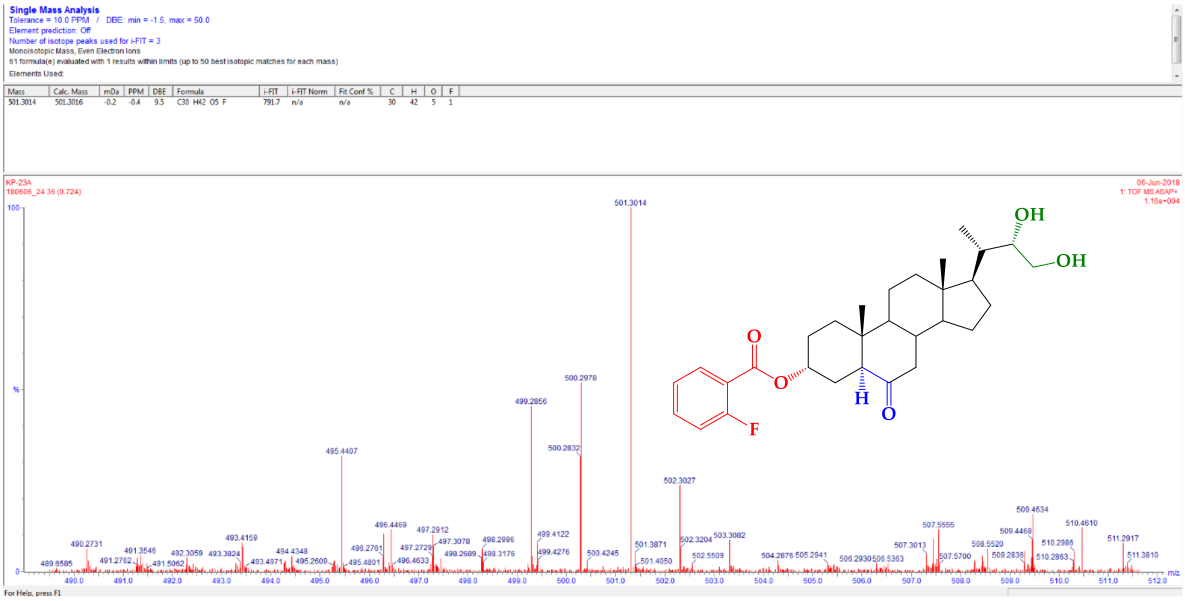

Figure S20: HRMS (API⁺) spectrum of (22*S*)-22,23-dihydroxy-6-oxo-24-nor-5α-cholan-3α-yl 2-fluorobenzoate (**19b**)
